# GreenHill: a de novo chromosome-level scaffolding and phasing tool using Hi-C

**DOI:** 10.1186/s13059-023-03006-8

**Published:** 2023-07-11

**Authors:** Shun Ouchi, Rei Kajitani, Takehiko Itoh

**Affiliations:** School of Life Science and Technology, Tokyo Institute of Technology, 2-12-1 Ookayama, Meguro-Ku, Tokyo, 152-8550 Japan

**Keywords:** Genome assembly, Haplotype, Hi-C, Scaffolding, Phasing

## Abstract

Chromosome-level haplotype-resolved genome assembly is an important resource in molecular biology. However, current de novo haplotype assemblers require parental data or reference genomes and often fail to provide chromosome-level results. We present GreenHill, a novel scaffolding and phasing tool that considers various assemblers’ contigs as input to reconstruct chromosome-level haplotypes using Hi-C without parental or reference data. Its unique functions include new error correction based on Hi-C contacts and the simultaneous use of Hi-C and long reads. Benchmarks reveal that GreenHill outperforms other approaches in contiguity and phasing accuracy, and the majority of chromosome arms are entirely phased.

**Supplementary Information:**

The online version contains supplementary material available at 10.1186/s13059-023-03006-8.

## Background

Most of the higher eukaryotic organisms are diploid and possess two copies of homologous chromosomes. A nucleotide sequence from one homologous chromosome (i.e., copy-specific) is called a haplotype, and high-quality haplotype-resolved reference genome sequences, with separately constructed homologous chromosomes, are fundamental resources for research in genomics, agriculture [1], medicine [2, 3], and other related biological fields [4]. For example, when completely phased haplotypes from 32 human individuals were examined, researchers discovered several unidentified structural variants in disease-associated regions [5]. The set of full-length haplotypes of all chromosomes provides lossless information about an individual diploid genome, which is the ultimate goal of genome assembly. In the initial genome projects targeting inbred model organisms, researchers did not have to consider haplotypes differences because the inbred samples had few heterozygous sites. This is not the case even for the human genome, much less for wild-type samples; however, draft genomes with mosaics of haplotypes (pseudo-haplotypes) were continuously used for these samples owing to technical limitations. Recently, the importance of haplotypes has been widely recognized, and the number of studies in which haplotypes are separately investigated is increasing. However, most genome assemblers neglect the differences between haplotypes and construct mosaic haplotypes that do not exist or possess only one haplotype sequence, thereby misleading biological interpretation in downstream analysis.

There are three main approaches for haplotype-resolved assembly. The first approach is trio binning [6–8] that attempts haplotype-specific assembly by identifying parent-specific *k*-mers and labeling the long reads with *k*-mers as haplotype-specific markers. TrioCanu [6] separates long reads into two independent haplotypes, assembles two separate reads independently and generates two haplotype genomes. Hifiasm [7] with the trio-binning mode generates a phased assembly by removing one-side allele-derived haplotype reads from the assembly graph. Trio binning approaches provide highly accurate phased assembly; however, they require parental data which are often unavailable for wild individuals. The second approach is reference-based haplotype reconstruction [9, 10] that generates a haplotype by mapping reads to an existing reference, calling variants, and resolving the chain relationships between variants. However, this approach requires a high-quality reference genome and relies on mapping-based variant calls, which may lead to errors. Particularly, in regions with high heterozygosity or structural variants, it is difficult to call variants based on the mapping of reads, and the tools often fail to reconstruct haplotypes. The third approach is a de novo assembly-based method [11, 12]. On the assembly graph, a branched structure called a bubble is formed in a region where there are differences between haplotypes. De novo assembly-based methods generate haplotypes by resolving the chain of bubbles from the information in the reads that link the bubbles. This approach can reconstruct genomes without parental data and references, and can capture variants that are not included in the reference genomes. Although long homozygous regions and repetitive sequences hinder haplotype assembly, the advent of long read DNA sequencing technologies overcome the limitations associated with these regions and enhance the length of assembled haplotypes.

To obtain longer chromosome-level haplotypes, chromosome conformation capture technologies, such as Hi-C [13] have been used in haplotype-aware assemblies [14–19]. FALCON-Phase [14] uses the results of FALCON-Unzip [11] as input and phases haplotypes using Hi-C. The Hi-C method can also be utilized for scaffolding, a process to determine the orders of assembled sequences; however, FALCON-Phase has no scaffolding function. Therefore, it is combined with other Hi-C scaffolding tools to reconstruct chromosome-level haplotypes. However, chromosomal-level haplotype assembly generated by FALCON-Phase has low accuracy and swaps large haplotype blocks between the two phases [20]. Like FALCON-Phase, Hifiasm Hi-C mode has a Hi-C phasing function [21] but does not use Hi-C data for scaffolding or resolving repeats. AllHiC [15, 16], a Hi-C scaffolding and phasing tool for polyploid genomes, requires a priori chromosome number and a closely related reference genome as input. As a fundamental problem, the accuracy of Hi-C scaffolders is often insufficient for automatically determining end-to-end chromosomal sequences. In the Vertebrate Genome Project, which intends to produce thousands of chromosome-level draft genomes, it was argued that manual corrections of sequences are necessary although Hi-C data are used [22]. This problem likely affects the haplotype phasing described above and demands a more accurate Hi-C scaffolder.

The existence of various types of data structures in assembler program results is another factor that hinders the completion of the genome sequence at the chromosome-level and subsequent use of the results for downstream analysis. Particularly, certain assemblers, such as Platanus-allee [12] and Hifiasm [7], output pairs of separated heterozygous regions in addition to collapsed homozygous regions that cannot be phased. FALCON-Unzip [11] outputs long pseudo-haplotype sequences (primary contigs) and phased short sequences (alternative contigs and haplotigs). Unfortunately, this result does not provide information on where the phased short sequences correspond to long pseudo-haplotype sequences. Moreover, the long pseudo-haplotype sequences were mosaic-like for both alleles. Furthermore, assembling a diploid genome using assemblers such as Canu [23], Flye [24], or SMARTdenovo [25] often produce contigs between one and two times the length of the haploid genome size, depending on the heterozygosity of the target genome. This is due to the fact that regions with high heterozygosity are assembled separately for each haploid, whereas regions with low heterozygosity are collapsed. In many cases, users have to identify and purge contigs derived from separately assembled heterozygous alleles [22] using external tools, such as Purge_dups [26]; however, these circumstances hamper the automation of genome assemblies.

In this study, we present GreenHill, a novel scaffolding and phasing tool using Hi-C in combination with other read information, such as long read, mate-pair, and paired-end reads (PE). GreenHill is designed to generate chromosomal-level haplotypes without parental data or references; in other words, it adopts a de novo assembly approach. Using a newly developed algorithm, long reads and Hi-C were synergistically used to improve the accuracy of the resulting haplotypes. In addition, this tool can automatically detect homologous regions in the input assembly (contigs) and handle any type of assembly, thereby enhancing its versatility. We evaluated the performance of GreenHill on de novo assemblies from both simulation data and actual data from a variety of species. Benchmarking results indicate that GreenHill outperforms other tools in various aspects, suggesting that this tool can facilitate the automation of de novo assembly of chromosome-level haplotypes.

## Results

### Overview of GreenHill

**Fig. 1:**
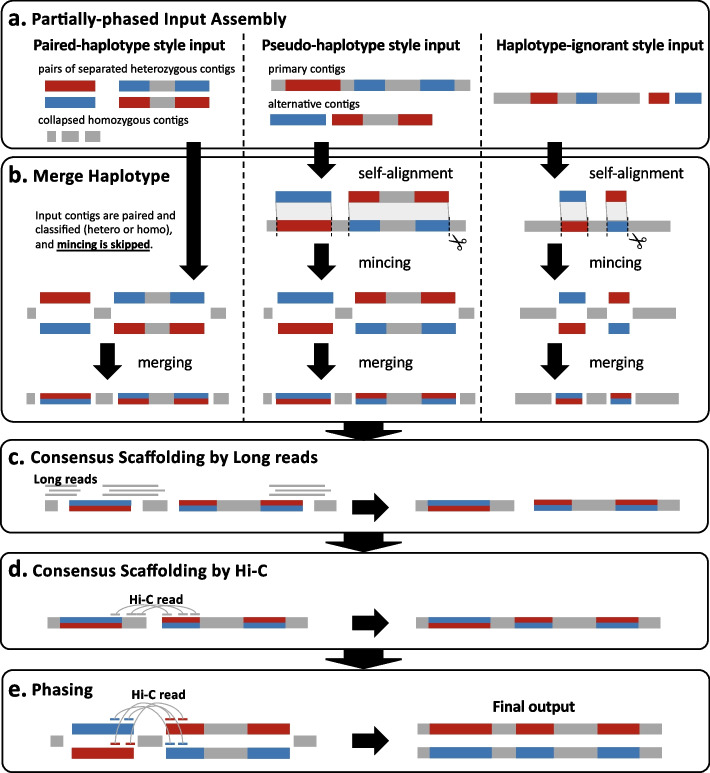
Overview of GreenHill. **a** GreenHill receives assembled contigs from other assembler as inputs. Any format of contigs is acceptable, such as paired-haplotype, pseudo-haplotype, and haplotype-ignorant styles. **b** Contig pairs that consist of the same loci from homologous chromosomes were identified, and each contig pair was merged into a single contig as consensus contig. **c** Consensus contigs were scaffolded using long reads. **d** Consensus contigs were scaffolded using Hi-C. **e** Consensus contigs were divided into two contigs and phased using long reads and Hi-C

An overview of GreenHill is shown in Fig. 1. The details on each step are present in the Methods section. GreenHill receives Hi-C and long reads in addition to the assembled contigs (output result sequences) from other assemblers as inputs, and simultaneously performs scaffolding and phasing. Any format of assembled sequences (contigs) is acceptable, such as (1) paired-haplotype (fully phased output: primary and secondary contigs), (2) pseudo-haplotype (separate output: consensus and alternative contig), and (3) haplotype-ignorant (mixed output of consensus and alternative contig) styles. The formats of Platanus-allee [12], FALCON-Unzip [11], and Canu [23] correspond to the aforementioned styles, respectively. First, contig pairs that consist of the same loci from homologous chromosomes were identified, and each corresponding pair was merged into a single contig as a “consensus contig” (Fig. 1b). The pair information and merged contigs were retained and reused in the downstream step. Next, consensus contigs were inputted into the scaffolding (Fig. 1c and d). As a unique function, this step is executed using both Hi-C and long reads, whereas the existing Hi-C scaffolders only accept one type of read. GreenHill utilizes long reads to improve the quality of scaffolds in various ways as follows: joining contigs, checking the joined contig pairs, and determining the breakpoints of misassemblies inferred using Hi-C data. In addition, the Hi-C scaffolding module has a characteristic error-detection function, which uses a variance-based thresholding method [27] for the contact map of Hi-C. Lastly, consensus contigs were divided into two contigs using the information saved in the merge haplotype step (Fig. 1b) and phased using both long read and Hi-C read links (Fig. 1e).

### Comparison of GreenHill with other approaches

We evaluated the performance of GreenHill and compared it with the results of other approaches employing FALCON-Phase and Hi-C scaffolding tools. We followed the procedure proposed in the FALCON-Phase paper [14], which involves running FALCON-Phase twice, before and after Hi-C scaffolding. First, input contigs were phased using FALCON-Phase. The input contigs were generated using multiple tools (in addition to FALCON-Unzip), and FALCON-Phase was used to extend the phased contigs. Canu's contigs were modified using Purge_dups [26] for FALCON-Phase. Next, contigs from FALCON-Phase were scaffolded using two existing state-of-the-art Hi-C scaffolding tools (SALSA2 [28] and 3D-DNA [29]) because FALCON-Phase lacks scaffolding function. After scaffolding, the scaffolds from Hi-C scaffolding tools were phased using FALCON-Phase and chromosome-level and haplotype-aware assemblies were generated. The details of the procedures above are described in the Methods section. In benchmarks with high-fidelity reads (HiFi reads [30]), Hifiasm Hi-C mode [21], which generates haplotype-resolved assembly using Hi-C, was also compared with GreenHill in addition to the combined pipelines using FALCON-Phase and Hi-C scaffolding tools.

### Assembly performance metrics

The assembly performance was evaluated by several metrics given below.

#### Contiguity

N50 was used to measure the contiguity of the assembly. N50 was defined as the length of the shortest contig for which longer and equal-length contigs covered at least 50% of the assembly.

#### Accuracy

Quality value (QV), switch error rate, and phasing accuracy were used to measure the accuracy of the assembly. QV was calculated using Merqury [31], assuming that *k*-mers are found only in the assembly as fine-scale errors. Switch error rates were calculated using Merqury with the haplotype-specific* k*-mer (hap-mer) found in the assembly. Phasing accuracy was calculated using an in-house script with hap-mers. Phasing accuracy was defined as follows:

$$\mathrm{Phasing}\;\mathrm{accuracy}=\frac{\mathrm{the}\;\mathrm{total}\;\mathrm{number}\;\mathrm{of}\;\mathrm{hap}-\mathrm{mers}\;\mathrm{from}\;\mathrm{the}\;\mathrm{major}\;\mathrm{haplotype}}{\mathrm{the}\;\mathrm{total}\;\mathrm{number}\;\mathrm{of}\;\mathrm{hap}-\mathrm{mers}\;\mathrm{from}\;\mathrm{both}\;\mathrm{haplotypes}}$$

This corresponds to the proportion of the majority of hap-mers in a scaffold. Notably, only one switch error can cause a large decrease in the value (e.g., error in the middle of a scaffold), and a low value of this indicator suggests large-scale haplotype inconsistency, which can have a negative effect on downstream analysis.

If the reference genome was available, we evaluated the accuracy of the assembly using the reference alignment-based method instead of the *k*-mer-based one (Merqury). The number of misassemblies and switch errors was calculated in a similar manner as previously described [12].

### Benchmarking experiments

We evaluated the performance of GreenHill on de novo assemblies using both simulation data and actual data from a variety of species.

#### *Caenorhabditis elegans* simulation dataset

We initially generated a simulated diploid dataset of *Caenorhabditis elegans* consisting of crosses of N2 and CB4856 strains (heterozygosity, 0.31%) for detailed accuracy evaluation (Additional file 1: Table S1). In this benchmark, assembled haplotypes were evaluated based on the reference genome, and the influence of local dense chromatin conformation, such as topologically associated domains (TAD) for Hi-C data, was excluded. We used ART [32] to generate Illumina paired-end reads for each strain at 80 × coverage, which was calculated for the haploid genome size. PacBio long reads (continuous long reads, CLR) were sequenced using Sequel and downsampled for each strain at 80 × coverage. Sim3C [33] (default setting except for the –linear option) was used to generate Hi-C reads for each strain at 60 × coverage. The reads from each strain were combined, resulting in a read set for the simulated diploid genome (N2 × CB4856). Three input style contigs were tested as follows: paired-haplotype (Platanus-allee), pseudo-haplotype (FALCON-Unzip), and haplotype-ignorant (Canu) style inputs.

Table 1 shows the output assembly statistics for the *C. elegans* data. FALCON-Unzip input results revealed that FALCON-Unzip + GreenHill achieved the largest N50 (17.1 Mb), while FALCON-Unzip + SALSA2 yielded fragmented assembly (N50: 9.5 Mb). Assembly accuracy indicated that FALCON-Unzip + GreenHill had the smallest number of switch errors and the second smallest number of misassemblies. FALCON-Unzip + 3D-DNA assembly had the largest number of misassemblies and switch errors. Canu input results revealed that Canu + GreenHill achieved the largest N50, smallest number of misassemblies, and smallest number of switch errors in comparison with the results from other scaffolding tools. However, Platanus-allee + GreenHill achieved the most accurate result in terms of switch errors. In summary, GreenHill generated the most accurate results for each style of contig, suggesting its versatility.

**Table 1:** Benchmarking results for *Caenorhabditis elegans* data

Assembler	Size (bp)	Max Length (bp)	N50 (bp)	#misassembly	#switch error
Platanus-allee + GreenHill	208,814,372	20,808,020	17,065,040	105	1,657
FALCON-Unzip + GreenHill	204,407,441	20,723,971	**17,142,644**	108	**2,121**
FALCON-Unzip + 3D-DNA	226,102,670	22,381,602	16,567,875	286	2,444
FALCON-Unzip + SALSA2	216,393,292	13,113,712	9,455,592	**104**	2,244
Canu + GreenHill	195,748,221	19,835,919	**16,219,706**	**182**	**1,742**
Canu + 3D-DNA	223,116,127	21,318,404	14,712,650	665	2,165
Canu + SALSA2	209,942,443	15,218,531	6,564,248	315	1,989

Size, N50, #misassembly, and #switch error were calculated for sequences whose length ≧500 bp. Size represents the size of assemblies generated by each assembler. #misassembly and #switch error is the number of misassemblies and switch errors calculated using the reference alignment-based method. A bold value indicates the best one for each input assembly

#### *Drosophila melanogaster* dataset

Subsequently, we tested whether GreenHill worked with HiFi reads using HiFi actual data and Hi-C simulation data of *D. melanogaster* (Additional file 1: Table S1). Haplotype-ignorant-style input contigs generated using HiCanu [34] and the haplotype-aware assembler for HiFi reads, Hifiasm [7], were tested. Haplotype-ignorant-style input contigs generated using Canu [23] (for CLR) were tested for comparison with HiFi input result. FALCON-Unzip was not tested because read data in an acceptable format (BAM files) were unavailable. Hi-C reads were simulated in a similar manner to those of *C. elegans* benchmarks. The target sample was an F1 individual from the ISO1 and A4 strains (heterozygosity, 0.74%) [34], and paired-end reads from its parents were used for evaluation. The number of misassemblies and switch errors was calculated using the reference genomes of the parental strains.

Table 2 indicates that the N50 values of GreenHill exceeded those of the other results in all cases. In each case, the number of misassemblies of GreenHill was approximately half than those of the other results. GreenHill had the smallest number of switch errors, except in one case (HiCanu input). These results suggest that GreenHill functions well for inputting either HiFi or CLR reads.

**Table 2:** Benchmarking results for *Drosophila melanogaster* data

Assembly	Input for contigs	Size (bp)	Max Length (bp)	N50 (bp)	#misassembly	#switch error
Canu + GreenHill	CLR	298,393,407	32,550,496	**25,267,681**	**515**	**2,316**
Canu + 3D-DNA		377,404,855	14,057,924	637,829	1,095	2,604
Canu + SALSA2		318,193,525	23,535,552	12,777,832	661	2,409
HiCanu + GreenHill	HiFi	321,609,584	33,217,621	**24,975,482**	**930**	686
HiCanu + 3D-DNA		351,304,469	21,680,562	2,482,127	1,631	688
HiCanu + SALSA2		297,968,925	28,602,144	24,843,359	1,435	**646**
Hifiasm + GreenHill	HiFi	307,145,492	27,892,039	**24,570,326**	**742**	**480**
Hifiasm + 3D-DNA		371,012,458	33,126,928	1,795,858	2,546	752
Hifiasm + SALSA2		327,819,782	25,140,693	9,592,386	2,330	735
Hifiasm Hi-C mode		308,439,591	26,052,400	21,522,312	1,346	637

Size, N50, #misassembly, and #switch error were calculated for sequences whose length ≧500 bp. Size represents the size of assemblies generated by each assembler. #misassembly and #switch error is the number of misassemblies and switch errors calculated using the reference alignment-based method. A bold value indicates the best one for each input assembly

#### Cow dataset

The third benchmark evaluated GreenHill’s performance on actual data using the actual cow (*Bos taurus*) dataset (Additional file 1: Table S1) with trio data from offspring and parents. Its heterozygosity is estimated to be 0.65–0.93%. The CLR, short (paired-end), and Hi-C reads from the offspring were used for de novo assembly and the parental short reads were used for evaluation. The estimated genome size was approximately 3 Gb; thus, these data were suitable for evaluating the performance of a relatively large genome. Platanus-allee was not used as input for GreenHill because the available paired-end read data were based on 2-channel SBS technology using NextSeq 500. The sequence quality was low and thus, Platanus-allee generated the assembly with a high switch error rate (Additional file 1: Table S2). In contrast, FALCON-Unzip generated contigs with a large N50 (4.6 Mb) and a low switch error rate (0.18), suggesting that the contigs are of high quality and are suitable for downstream analysis.

Table 3 shows the output assembly statistics for the cow dataset. FALCON-Unzip + GreenHill achieved the largest N50 (89.8 Mb), outperforming the other approaches. In addition, FALCON-Unzip + GreenHill had the highest QV, lowest switch error rate, and highest phasing accuracy.

**Table 3:** Benchmarking results for cow data

Assembler	Size (bp)	Max Length (bp)	N50 (bp)	QV	Switch error rate	Phasing accuracy
FALCON-Unzip + GreenHill	5,265,512,220	156,630,926	**89,758,138**	**42.40**	**0.18**	**0.949**
FALCON-Unzip + 3D-DNA	5,649,125,145	156,722,321	77,197,018	41.15	0.23	0.826
FALCON-Unzip + SALSA2	5,479,993,517	139,927,026	68,805,579	41.25	0.22	0.856

Size and N50 were calculated for sequences whose length ≥ 500 bp. Size represents the size of assemblies generated by each assembler. QV and switch error rate were calculated by Merqury. Phasing accuracy represents the proportion of the majority of hap-mers in a scaffold, and a high value of this indicator suggests large-scale haplotype consistency. A bold value indicates the best one for each input assembly

**Fig. 2:**
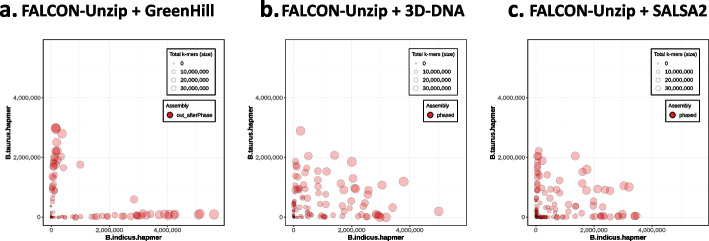
Hap-mer blob plots for cow data. The numbers of hap-mers (parent-specific *k*-mer) from the mother and father are indicated on the x-axis and y-axis, respectively. Each scaffold is represented as a circle. The sizes of the circles indicate sequence lengths. The higher the phasing accuracy, the closer the circles are aligned to the axis. **a** FALCON-Unzip + GreenHill result; **b** FALCON-Unzip + 3D-DNA result; **c** FALCON-Unzip + SALSA2 result

To gain additional insight regarding the phasing accuracy of the assemblies, we visualized the compositions of hap-mers and lengths of scaffolds from hap-mer blob plots using Merqury (Fig. 2). Each scaffold is represented by a circle, whose size indicates the length of the scaffold. The number of hap-mers from the mother and father is indicated on the x-axis and y-axis, respectively. The higher the phasing accuracy, the fewer the number of hap-mers from the other haplotype; therefore, the circles are aligned closer to the axis. The circles in the hap-mer blob plot of FALCON-Unzip + GreenHill were aligned closer to the axis than those of the FALCON-Phase-based approaches, indicating that FALCON-Unzip + GreenHill outperformed FALCON-Phase-based approaches in phasing accuracy.

**Fig. 3:**
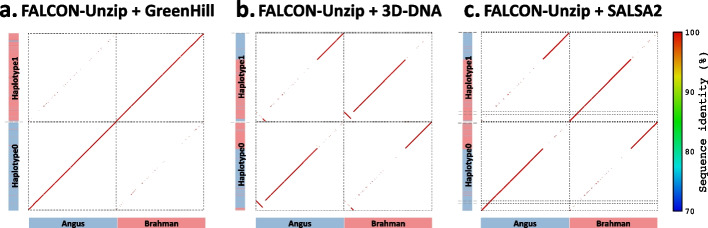
Alignment dot plots for the cow scaffolds on the Chromosome 1. Dot plots of alignment between benchmark results and trio-binned assembly for the cow scaffolds on the Chromosome 1. Each dot in the plot represents an alignment between benchmark results and the trio-binned assembly, and the color indicates the identity of the alignment. The upper left and lower right quadrants correspond to the scaffolds, which are color-coded according to their haplotypes. Red, blue, and gray correspond to maternal, paternal, and homozygous regions, respectively. The black dashed lines represent the boundaries of the scaffolds. **a** FALCON-Unzip + GreenHill result; **b** FALCON-Unzip + 3D-DNA result; **c** FALCON-Unzip + SALSA2 result

To further validate the correctness of the assemblies, we created alignment dot plots using nucmer, delta-filter, and a modified version of the mummerplot in the MUMmer package [35] to display the consistency between the benchmark results and the trio-binned assembly (TrioCanu). Figure 3 shows the alignment dot plots for Chromosome 1. FALCON-Phase-based approaches had large switch errors that swap large haplotype blocks between two haplotypes, while FALCON-Unzip + 3D-DNA had an inversion error. In contrast, FALCON-Unzip + GreenHill did not exhibit large switch errors or misassemblies. Assembly contiguity revealed that FALCON-Unzip + SALSA2 reconstructed each Chromosome 1 haplotype with multiple scaffolds (> 1 Mb), whereas FALCON-Unzip + GreenHill reconstructed each Chromosome 1 haplotype with a single scaffold (> 1 Mb).

#### Zebra finch dataset

Fourth, we performed a benchmark using the actual dataset of Zebra finches (*Taeniopygia guttata*), which had trio data with a high heterozygous sample (1.47%) (Additional file 1: Table S1). The CLR, HiFi, 10X, and Hi-C reads from the offspring were used for the de novo assembly, and the parental short reads were used for evaluation. Contig assembly was performed using Platanus-allee (for 10X and CLR), FALCON-Unzip (for CLR), Canu (for CLR), and Hifiasm (for HiFi). We used 10X data instead of paired-end data for running Platanus-allee because paired-end reads were unavailable. Unlike the results from the cow dataset, the N50 length of the contigs using FALCON-Unzip was relatively small (931 kb; Additional file 1: Table S2), probably due to its high heterozygosity. We anticipate that the performance of scaffolders for fragmented inputs can be measured using this benchmark.

The output assembly statistics for the zebra finch data are listed in Table 4. FALCON-Unzip input results indicated that FALCON-Unzip + GreenHill generated scaffolds with similar N50 lengths (70.6 Mb) in comparison with those generated using FALCON-Unzip + 3D-DNA (N50: 73.4 Mb). FALCON-Unzip + SALSA2 yielded a considerably fragmented assembly (N50: 14.2 Mb). Assembly accuracy using GreenHill outperformed the other approaches in QV, switch error rate, and phasing accuracy. Canu input results revealed that GreenHill generated scaffolds with similar N50 lengths (70.9 Mb) as 3D-DNA (74.6 Mb) and achieved the highest QV and phasing accuracy. Of all the combinations of the contig-assemblers and scaffolders with CLR, Platanus-allee + GreenHill achieved the highest phasing accuracy. The results of the contigs obtained using Canu had a higher switch error rate and lower phasing accuracy than those obtained from other tools, suggesting that haplotype-ignorant style input contigs are relatively difficult to handle for phasing. Nonetheless, GreenHill showed the best performance in accuracy for each input contig set, which supports its versatility for input contig. Hifiasm input results showed that GreenHill generated scaffolds with the largest N50 lengths (62.8 Mb), while other tools generated more fragmented scaffolds (N50 < 12 Mb). Hifiasm Hi-C mode generated the most accurate results in terms of QV, switch error rate, and phasing accuracy, but the result was more fragmented than GreenHill due to the lack of a scaffolding function. Moreover, GreenHill generated scaffolds with high phasing accuracy (> 0.9) despite it having high contiguity. Consequently, Greenhill’s assemblies were also generated with good accuracy and the highest contiguity for Hifiasm input results, suggesting its robustness to HiFi data.

**Table 4:** Benchmarking results for zebra finch data

Assembler	Input for contigs	Size (bp)	Max Length (bp)	N50 (bp)	QV	Switch error rate	Phasing accuracy
Platanus-allee + GreenHill	10X, CLR	2,309,934,319	152,477,488	61,881,567	35.55	0.57	0.953
FALCON-Unzip + GreenHill	CLR	2,025,894,925	150,748,938	70,617,212	**35.97**	**0.79**	**0.886**
FALCON-Unzip + 3D-DNA		2,165,277,869	153,652,046	**73,366,558**	35.29	0.87	0.639
FALCON-Unzip + SALSA2		2,153,682,369	67,065,260	14,220,490	35.35	0.87	0.717
Canu + GreenHill	CLR	1,995,878,168	148,688,728	70,920,789	**35.98**	2.28	**0.849**
Canu + 3D-DNA		2,283,995,575	163,319,697	**74,641,957**	35.57	2.22	0.589
Canu + SALSA2		2,263,303,629	27,304,387	6,544,809	35.61	**2.22**	0.658
Hifiasm + GreenHill	HiFi	2,139,611,083	152,614,629	**62,848,794**	49.40	0.02	0.914
Hifiasm + 3D-DNA		2,667,759,650	77,682,004	1,858,283	49.83	0.02	0.929
Hifiasm + SALSA2		2,352,582,037	64,427,564	11,305,740	49.84	0.02	0.784
Hifiasm Hi-C mode		2,172,318,724	33,557,543	7,896,913	**50.51**	**0.01**	**0.997**

Size and N50 were calculated for sequences whose length ≥ 500 bp. Size represents the size of assemblies generated by each assembler. QV and switch error rate were calculated by Merqury. Phasing accuracy represents the proportion of the majority of hap-mers in a scaffold, and a high value of this indicator suggests large-scale haplotype consistency. A bold value indicates the best one for each input assembly

**Fig. 4:**
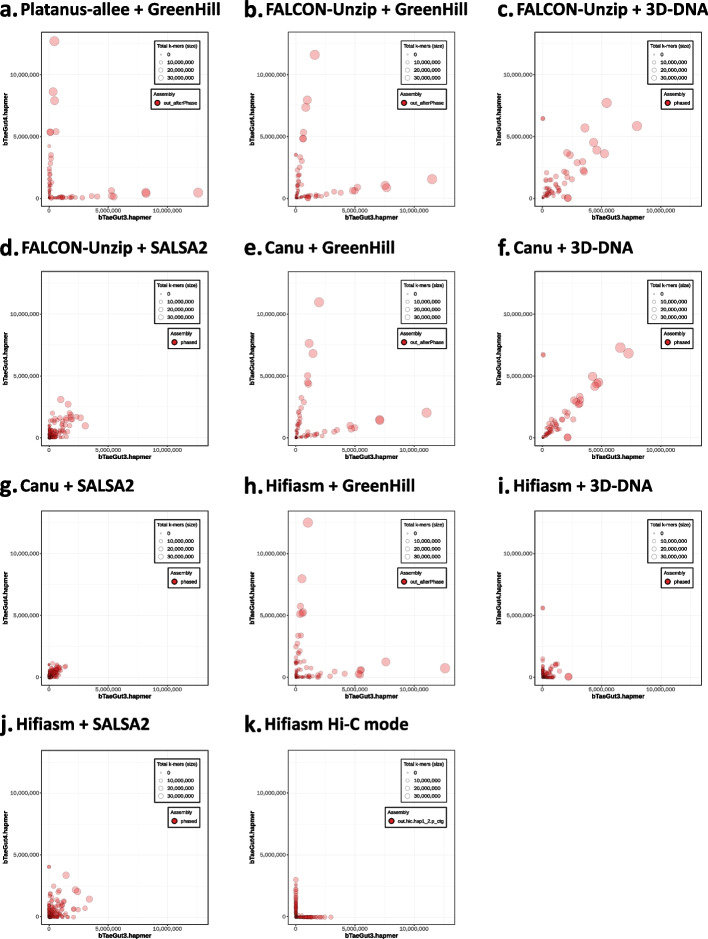
Hap-mer blob plots for zebra finch data. The numbers of hap-mers (parent-specific *k*-mer) from the mother and father are indicated on the x-axis and y-axis, respectively. The sizes of the circles indicate sequence lengths. The higher the phasing accuracy, the closer the circles are aligned to the axis. **a** Platanus-allee + GreenHill; **b** FALCON-Unzip + GreenHill result; **c** FALCON-Unzip + 3D-DNA result; **d** FALCON-Unzip + SALSA2 result; **e** Canu + GreenHill result; **f** Canu + 3D-DNA result; **g** Canu + SALSA2 result; **h** Hifiasm + GreenHill result; **i** Hifiasm + 3D-DNA result; **j** Hifiasm + SALSA2 result; **k** Hifiasm Hi-C mode result

**Fig. 5:**
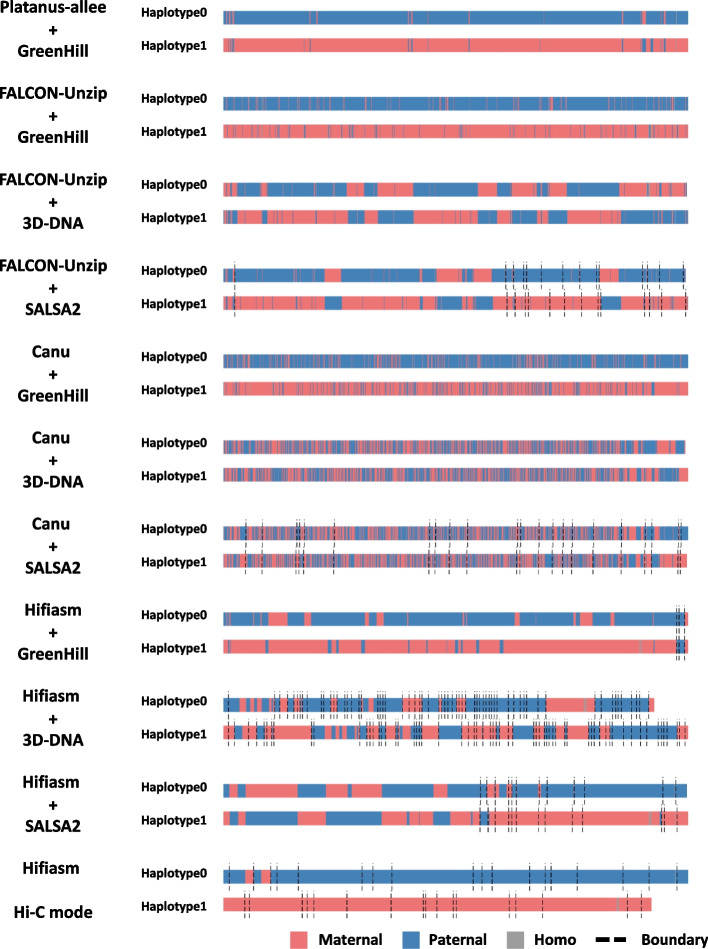
Haplotype structures of the zebra finch scaffolds on the Chromosome 3. Each scaffold was color-coded according to the corresponding haplotype. Red, blue, and gray regions correspond to maternal, paternal, and homozygous ones, respectively. The black dashed lines represent boundaries between scaffolds

In addition, the hap-mer blob plots of each output assembly show that GreenHill-based assemblies have greater phasing accuracy than FALCON-phase-based assemblies (Fig. 4). To validate the accuracy at the chromosomal level, we color-coded scaffolds according to the corresponding haplotype using a hap-mer (Fig. 5, Additional file 1: Fig. S1). Figure 5 shows the haplotype structure of Chromosome 3. Although FALCON-Phase-based approaches exhibited several large switch errors, GreenHill-based approaches generated each haplotype on Chromosome 3 as a single scaffold (> 1 Mb) without large switch errors (except for the Hifiasm input result).

#### Other species datasets

To further investigate GreenHill’s versatility, we used the tools for genome assembly construction for three species: budgerigar (*Melopsittacus undulatus*), black rhinoceros (*Diceros bicornis*), and sterlet (*Acipenser ruthenus*). The estimated heterozygosities were 1.04%, 0.21%, and 0.58%, respectively. The sequencing data are publicly available (Additional file 1: Table S1). The long reads (CLR or HiFi) and Hi-C reads from offspring were used for de novo assembly and the parental short reads were used for evaluation. Input contigs were generated using FALCON-Unzip (for budgerigar) and Hifiasm (for black rhinoceros and sterlet).

These results are shown in Table 5. GreenHill yielded scaffolds with similar (budgerigar: 88.1 Mb) or larger (black rhinoceros: 52.2 Mb, sterlet: 25.8 Mb) N50 lengths compared to other tools. Hifiasm Hi-C mode generated the most accurate results, but they were more fragmented than those of GreenHill due to its lack of a scaffolding function. GreenHill generated more accurate results than FALCON-Phase-based approaches in terms of switch error rate and phasing accuracy, except in cases where the N50 values of the FALCON-Phase-based approaches were very low (< 4 Mb). Furthermore, the hap-mer blob plots for each output assembly indicated that the phasing accuracy of GreenHill-based assemblies is slightly lower than that of Hifiasm Hi-C mode assemblies but higher than that of FALCON-Phase-based assemblies (Additional file 1: Fig. S2, S3, S4). These results show GreenHill’s versatility for use in different species.

**Table 5:** Benchmarking results for other species

Species	Assembler	Input for contigs	Size (bp)	Max Length (bp)	N50 (bp)	QV	Switch error rate	Phasing accuracy
Budgerigar	FALCON-Unzip + GreenHill	CLR	2,273,118,342	140,405,933	88,135,996	**40.78**	**0.28**	**0.896**
	FALCON-Unzip + 3D-DNA		2,620,388,952	159,155,647	**90,074,506**	39.20	0.36	0.701
	FALCON-Unzip + SALSA2		2,437,415,698	95,254,517	34,574,245	39.31	0.34	0.741
Black rhinoceros	Hifiasm + GreenHill	HiFi	5,325,705,542	101,752,101	**52,259,952**	58.44	0.05	0.982
	Hifiasm + 3D-DNA		6,523,963,142	136,733,613	48,162,421	55.16	0.09	0.615
	Hifiasm + SALSA2		6,206,904,826	40,586,421	3,069,332	55.53	**0.05**	0.785
	Hifiasm Hi-C mode		6,047,793,056	94,248,160	30,503,132	**67.13**	0.06	**0.995**
Sterlet	Hifiasm + GreenHill	HiFi	3,712,891,508	83,435,872	**25,813,405**	58.82	0.03	0.849
	Hifiasm + 3D-DNA		3,882,750,516	14,958,612	1,019,399	54.98	0.04	0.889
	Hifiasm + SALSA2		3,474,135,770	47,228,216	7,128,235	55.15	0.04	0.751
	Hifiasm Hi-C mode		3,748,196,843	55,271,118	9,593,284	**60.99**	**0.02**	**0.975**

Size and N50 were calculated for sequences whose length ≥ 500 bp. Size represents the size of assemblies generated by each assembler. QV and switch error rate were calculated by Merqury. Phasing accuracy represents the proportion of the majority of hap-mers in a scaffold, and a high value of this indicator suggests large-scale haplotype consistency. A bold value indicates the best one for each input assembly

### Knock-out tests to evaluate the effectiveness of GreenHill

To confirm the effectiveness of the characteristic functions of GreenHill, we conducted tests in which certain functions were deactivated (knocked out). The target functions were simultaneous long-read-use (LR-use) and Hi-C-based corrections. The number of misassemblies was calculated using the reference genomes of parental strains for the *C. elegans* and *D. melanogaster* data and the trio-binned assembly for the cow and zebra finch data. As expected, the number of misassemblies and/or phasing accuracies dramatically worsened when the functions were knocked out (Additional file 1: Table S3), confirming the effectiveness of the novel functions.

### Runtime and memory usage evaluation

We evaluated the runtime and memory usage of the three tools on all datasets. Notably, GreenHill performs purging input contigs, mapping Hi-C reads to contigs, scaffolding with Hi-C, and phasing with Hi-C in the tool, so FALCON-Phase + Hi-C scaffolding tool’s runtime is calculated by adding the runtime of purge_dups, Hi-C mapping (Juicer [36] or Arima mapping pipeline [37]), Hi-C scaffolding (3D-DNA or SALSA2), and Hi-C phasing (FALCON-Phase). CPU times, real times, and peak memory usage of the tools were measured with the GNU time command on a computer with an Intel(R) Xeon(R) Gold 6342 CPU (2.80 GHz clocks, dual 24 cores) and 512 GB of RAM. The number of threads was specified as 48 for each process provided it was configurable.

Results are shown in Additional file 1: Table S4. GreenHill required similar or less time than other approaches. GreenHill generated assemblies within approximately 1 h for data with small genome sizes (< 500 Mb, *C. elegans* and *D. melanogaster*), and within 2 days for data with large genome sizes (> 2 Gb, Black rhinoceros). FALCON-Phase + 3D-DNA and FALCON-Phase + SALSA2 are time-consuming, especially when the genome size is large (approximately 10 days or more for black rhinoceros data). This may be due to the Hi-C mapping each time the Hi-C scaffolding tool and Hi-C phasing tool are run. GreenHill's peak memory usage was higher than the other tools, but it was less than 206 GB even for the data from large genome sizes (> 2 Gb, Black rhinoceros).

## Discussion

In this study, we developed a novel algorithm, GreenHill, and tested its performance and versatility using data from many species with various types of input contigs. In the first (*C. elegans*) and the second (*D. melanogaster*) benchmark with the Hi-C simulated data, we confirmed that the basic performance of GreenHill was high under the condition of ideal Hi-C reads without local dense chromatin conformations, such as TAD, that can disturb the behaviors of Hi-C scaffolders. The high performance of GreenHill was demonstrated in the two benchmarks, one using CLR input and the other using HiFi input.

In the third (cow) and the fourth (zebra finch) benchmarks of actual data, the CLR input contigs were contiguous and relatively fragmented, respectively. Short contigs are difficult to determine the order and orient owing to the small number of mapped Hi-C reads. Therefore, fragmented contigs may be difficult for the scaffolders. GreenHill achieved the highest values for all metrics regarding phasing quality for both contiguous and fragmented input contigs, suggesting its versatility. It is worth noting that 3D-DNA and SALSA2 had substantially low phasing accuracy (maximum of 0.639 and 0.717, respectively) for the zebra finch, which implies that approximately one-third of regions in their resulting haplotype sequences were misphased. Moreover, GreenHill exhibited phasing accuracies ranging from 0.849–0.953 in both cow and zebra finch benchmarks. The results of Platanus-allee + GreenHill showed high phasing accuracy (0.953 for zebra finch), and this combination may be preferable for accurate haplotypes if a short-read library has sufficient quality and coverage. In addition, the majority of phased scaffolds from GreenHill covered the entire chromosome arm. Therefore, the result of GreenHill was close to our goal of de novo assembly of haplotypes of all chromosomes.

We tested GreenHill’s performance on a variety of heterozygosity species ranging from 0.21 ~ 1.47. The heterozygosity of a genome assembly can have a significant impact on its quality. High levels of heterozygosity can lead to increased fragmentation and misassembly, while low levels of heterozygosity can make phasing difficult with few heterozygous sites. In all data, GreenHill was able to construct highly accurate and contiguous haplotypes and showed high robustness to heterozygosity.

Regarding algorithms, the unique functions of GreenHill include the simultaneous use of long and Hi-C reads and error correction using Hi-C contact information with variance-based threshold selection [27]. Some existing pipelines [14, 20] and protocols [22] accept both long and Hi-C reads; however, they separately use each read set. In addition, GreenHill iteratively attempts to scaffold and phase changing parameters such as *L* (see “ Consensus scaffolding using Hi-C” in the Methods section), which may be minor in terms of novelty but effective in improving the lengths of sequences. The effectiveness of the novel functions was confirmed in the benchmarks in which these functions were deactivated (knocked out).

GreenHill can accept fully or partially phased contigs using any tool. Moreover, even the haplotype-ignorant style contigs from Canu, which were assumed to be difficult to handle because contigs were not classified into primary and alternative phases, could be used to construct haplotypes, supporting the versatility of GreenHill. For the other types of input data in this study, we dealt with HiFi reads [30] which have low error rates (< 1%) as well as PacBio CLR whose error rates were high (10%–15%). The benchmark results with HiFi + Hi-C dataset (*D. melanogaster*, zebra finch, black rhinoceros, and sterlet) show that GreenHill also performs well on HiFi reads. HiFi reads may be more effective for phase haplotypes than CLR because of sequence accuracy. However, the DNA extraction protocol for HiFi reads requires a narrow range of fragment lengths (e.g., 15–18 kb) [30], whereas that for CLR can utilize all long fragments (e.g., ≥ 15 kb). Thus, it is possible that the HiFi protocol requires higher total DNA and is difficult to apply to samples in which DNA extraction is complex. This suggests that CLR has wider applicability than HiFi, and our benchmark results show high performance even for the CLR input case, which is more computationally challenging and versatile. In summary, GreenHill has versatility for both HiFi and CLR input contig types and works well efficiently with high-error-rate long reads, suggesting the practicability of various projects targeting diploid organisms.

However, there are certain limitations of the study: (1) GreenHill has no function in detecting and correcting switch errors in the input assembly; therefore, the phasing accuracy of the assembly generated by GreenHill depends on the phasing accuracy of the input assembly. (2) GreenHill is designed for diploid genomes; therefore, it cannot handle polypoid genomes. Future studies should detect switch errors in the input assembly from mapping results of reads and extend a method to accommodate polyploid genomes.

## Conclusions

In this study, we presented GreenHill, a novel scaffolding and phasing tool for reconstructing chromosome-level haplotypes using Hi-C. Benchmarking with simulation and actual data showed that GreenHill outperformed other existing approaches in assembly contiguity and phasing accuracy. GreenHill, which can reconstruct chromosome-level haplotypes with high accuracy, is expected to facilitate a large variety of downstream analyses, such as structural variation analysis or gene analysis between haplotypes.

## Methods

### GreenHill method

GreenHill has three stages: (1) merging of two corresponding haplotigs into a “consensus contig,” (2) consensus scaffolding, and (3) phasing (Fig. 1). GreenHill was implemented in an easy-to-use open-source tool https://github.com/ShunOuchi/GreenHill.

### Input assembly

GreenHill inputs a partially phased assembly generated by the other assemblers. “paired-haplotype style,” “pseudo-haplotype style,” and “haplotype-ignorant style” can be used as the input format.

The paired-haplotype style is the format in which a heterozygous haplotype contig is associated with a homologous counterpart from end to end as a bubble. For each bubble, the contig with more non-N bases is defined as a “primary bubble,” whereas the other contig is defined as a “secondary bubble.” A contig that is not a bubble is defined as a “non-bubble.” A paired-haplotype style was used for the Platanus-allee assembly.

The pseudo-haplotype style is the format consisting of pseudo-haplotype and alternative sequences. A pseudo-haplotype sequence contains collapsed regions with low or no heterozygosity and/or heterozygous regions. An alternative sequence is a heterozygous sequence that is associated with a pseudo-haplotype region. The pseudo-haplotype style is used for assemblers namely FALCON-Unzip.

The haplotype-ignorant style is the format consisting of sequences with no haplotype information. Using assemblers, such as Canu, certain highly heterozygous regions are unintentionally phased, resulting in assembly sizes 1–2 times the genome size. GreenHill can use such inputs.

### Merge haplotype

In the merge haplotype step, we identified the heterozygous regions in the input assembly. The merge haplotype method depends on the input format.

For pseudo-haplotype and haplotype-ignorant style inputs, we exchanged the input format for the paired-haplotype style as follows (Additional file 1: Fig. S5a). First, we calculated the self-alignment of the input contigs using Minimap2 [38] (2.24-r1122) with the following options: “-D –secondary = no”. Alignment results with < 80% identity were filtered. Next, we computed the opposite contig for each contig, where the opposite contig was the contig with the longest alignment to the contig. In order of alignment length, we associated a contig with the opposite contig. If the opposite contig of a contig *u* is already associated with another contig *v* and the alignment of *u* and that of* v* overlap, the contig *u* is removed as a repeat contig. We then mince contigs according to the opposite contig information. Lastly, the coverage and bubble information of the contigs were included in the header. The coverage of the contigs associated with another contig was set to *C*, and the coverage of other contigs was set to 2 × *C* (*C* is the constant value, default: 40).

For the paired-haplotype style (Platanus-allee) input, we first retrieved coverage depth and bubble information of contigs in the headers of the input assembly. Second, a primary-bubble contig and a secondary-bubble counterpart were merged into a consensus contig using bubble information (Additional file 1: Fig. S5b). For non-bubble contigs, if the coverage depth of a contig was < *C*_hetero_ × *r*_upper-threshold_, the contig was considered to be a heterozygous contig that could not be associated. Otherwise, the contig was considered a homozygous contig. *C*_hetero_ is the average coverage of heterozygous contigs calculated in a similar manner to that calculated by Platanus-allee (*r*_upper-threshold_ is a constant value; default = 1.75).

The merging result is stored in array *T*. A consensus contig that merges primary-bubble contig *u* and secondary-bubble contig *v* is stored as [*u*, *v*]. A heterozygous non-bubble contig *u* is stored as [*u*, -]. A homozygous non-bubble contig *u* is stored as [*u*, *u*].

### Mapping reads to contigs

For short reads (Illumina, 10X), the reads were mapped in a similar manner to that mapped by Platanus-allee using the *k*-mer exact unique match between a read and contig. The difference in GreenHill from Platanus-allee is as follows: if a k-mer matches two contigs and the contigs belong to the same consensus contig, the read is mapped on one of the contigs as a “consensus link.” Platanus-allee uses only unique *k*-mer matches for mapping. Consensus links are used in consensus scaffolding and not in phasing.

Long reads (PacBio) were mapped to contigs in a similar manner as that mapped by Platanus-allee using Minimap2. The difference in GreenHill from Platanus-allee is the method of greedy selecting the alignments. Platanus-allee selects the alignments according to the order of #match-sites, whereas GreenHill selects according to the order of sequence identity (#match-sites divided by alignment length).

For Hi-C reads, the reads were mapped in the same manner as the mapping short reads, except for ignoring the insert length and read orientation between pairs of reads.

### Consensus scaffolding using long reads

In the first step of consensus scaffolding, we scaffolded consensus contigs using long read (and PE) information in a similar manner as that used by Platanus-allee (Additional file 1: Fig. S6). The extension is that GreenHill can use Hi-C data to detect and correct misassemblies.

Briefly, we constructed a scaffold graph, where nodes represent contigs and edges indicate long read links between contigs. Next, we combined the non-branching nodes in which the indegree and outdegree are equal to one. Then, we removed erroneous edges using Hi-C (see the “ Detection of erroneous edges using Hi-C” section), and constructed a scaffold using an algorithm similar to that used in Platanus-allee.

### Consensus scaffolding using Hi-C

In the second step of consensus scaffolding, we also extended consensus scaffolds (results from the previous step) using Hi-C information (Additional file 1: Fig. S7). Chromosome-level consensus scaffolds were generated as follows.

1. We detected and corrected misassemblies in the input consensus scaffold. First, we detected misassembly candidate regions in the consensus scaffold using Hi-C (see “Detection of misassembly using Hi-C”), and determined the breakpoints using the coverage of long read (and PE). Since a drop in coverage indicated a likely misassembly, we divided the scaffold at a minimum-coverage point in each misassembly-candidate region.
2. We constructed an undirected graph where nodes represented the 5ʹ or 3ʹ ends of scaffolds and edges indicated Hi-C links between the ends of scaffolds. Node *v*’ was denoted as the opposite end of node* v*, and the edge weight was calculated by the number of the Hi-C read pairs bridging the input scaffolds in the region at a distance of *L* from the ends. Edges with weights smaller than a predefined threshold (default: 50 [L < 100 kb], 100 [L ≥ 100 kb]) were filtered out.
3. We identified the edge *e* with the maximum weight.
4. We checked if the edge *e* is an erroneous edge using Hi-C (see “ Detection of erroneous edges using Hi-C”) and long read (and PE) information (see “ Detection of erroneous edges using long reads”).
5. If edge *e* was surmised to be an erroneous edge at (4), it was removed; otherwise, we connected the start and end nodes of edge *e*.
6. We iterated procedures (3)–(5) until the graph remained constant.
7. We iterated procedures (2)–(6) and gradually increased *L* by 10 kb (i.e., from 10 to 100 kb) and 100 kb (i.e., from 100 kb to 1 Mb).
8. Without checking using long read (and PE) information, we reiterated procedures (2)–(7) to connect scaffolds across long repeat regions that cannot be exceeded by long read.

### Detection of erroneous edges using Hi-C

We checked the edge *e* between nodes *u* and *w* using the Hi-C contact matrix (Additional file 1: Fig. S8) based on two facts: (1) the intra-chromosomal Hi-C interaction frequency is higher than the inter-chromosomal interaction frequency and (2) the closer the distance, the stronger the intra-chromosomal interaction frequency [13].

1. We connected the scaffolds, represented as nodes *u* and *w* into a scaffold, and partitioned the scaffold into bins of fixed length (default: 100 kb). We created a Hi-C matrix *M* by counting the number of linking Hi-C read pairs between bins. Each pixel ${M}_{i,j}$ corresponds to the number of Hi-C links between a pair of bins (in this case, *i*^th^ and *j*^th^ bins).
2. We calculated separation scores *S* and *R* at the boundary between *u* and *w*. The separation scores *S* and *R* were defined as follows:
  1 $${\varvec{S}}=\boldsymbol{ }\sum\nolimits_{{\varvec{k}}}\frac{{{\varvec{N}}}_{{\varvec{i}}{\varvec{n}},{\varvec{k}}}{{\varvec{N}}}_{{\varvec{o}}{\varvec{u}}{\varvec{t}},{\varvec{k}}}{\left({{\varvec{\mu}}}_{{\varvec{i}}{\varvec{n}},{\varvec{k}}}-{{\varvec{\mu}}}_{{\varvec{o}}{\varvec{u}}{\varvec{t}},{\varvec{k}}}\right)}^{2}}{{\left({{\varvec{N}}}_{{\varvec{i}}{\varvec{n}},{\varvec{k}}}+{{\varvec{N}}}_{{\varvec{o}}{\varvec{u}}{\varvec{t}},{\varvec{k}}}\right)}^{2}}$$
  2 $${\varvec{R}}=\boldsymbol{ }\frac{\sum_{{\varvec{k}}}{{\varvec{L}}}_{{\varvec{o}}{\varvec{u}}{\varvec{t}},{\varvec{k}}}}{\sum_{{\varvec{k}}}{{\varvec{N}}}_{{\varvec{o}}{\varvec{u}}{\varvec{t}},{\varvec{k}}}}$$

where *k* is the distance from the diagonal; ${N}_{in,k}$ is the number of pixels in the inner region within *k* of the diagonal; ${N}_{out,k}$ is the number of pixels in the outer region at a distance *k* from the diagonal; ${\mu }_{in,k}$ is the average of the values of the pixels in the inner region at a distance *k* from the diagonal; ${\mu }_{out,k}$ is the average of the values of the pixels in the outer region at a distance *k* from the diagonal; and ${L}_{out,k}$ is the number of pixels whose value is lower than ${\mu }_{in,k}$ in the outer region at a distance *k* from the diagonal. The separation score *S* corresponds to the between-class variance, where the inner and outer regions are assumed to be two classes. This concept was derived from Otsu's method [27]. The other score *R* was used to handle the sparse contact map, in which misassemblies are falsely detected. If edge *e* is an erroneous edge, the difference between the inner and outer regions will be large, and the values of *S* and *R* will be large. *S* and *R* were calculated on both the upper and lower sides, and were denoted as *S*_upper_, *S*_lower_, *R*_upper_, and *R*_lower_.

(3) Edge *e* was surmised to be an erroneous edge if

3 $${{\varvec{S}}}_{\mathbf{u}\mathbf{p}\mathbf{p}\mathbf{e}\mathbf{r}}/{{\varvec{S}}}_{\mathbf{u}\mathbf{p}\mathbf{p}\mathbf{e}\mathbf{r},\boldsymbol{ }0}+\boldsymbol{ }{{\varvec{S}}}_{\mathbf{l}\mathbf{o}\mathbf{w}\mathbf{e}\mathbf{r}}/{{\varvec{S}}}_{\mathbf{l}\mathbf{o}\mathbf{w}\mathbf{e}\mathbf{r},\boldsymbol{ }0}\boldsymbol{ }\boldsymbol{ }\ge {\varvec{s}}\left(\mathbf{c}\mathbf{o}\mathbf{n}\mathbf{s}\mathbf{t}\mathbf{a}\mathbf{n}\mathbf{t}\mathbf{v}\mathbf{a}\mathbf{l}\mathbf{u}\mathbf{e};\mathbf{d}\mathbf{e}\mathbf{f}\mathbf{a}\mathbf{u}\mathbf{l}\mathbf{t},1.0\right)$$

and

4 $${{\varvec{R}}}_{\mathbf{u}\mathbf{p}\mathbf{p}\mathbf{e}\mathbf{r}}+{{\varvec{R}}}_{\mathbf{l}\mathbf{o}\mathbf{w}\mathbf{e}\mathbf{r}}\boldsymbol{ }\ge {\varvec{r}}\left(\mathbf{c}\mathbf{o}\mathbf{n}\mathbf{s}\mathbf{t}\mathbf{a}\mathbf{n}\mathbf{t}\mathbf{v}\mathbf{a}\mathbf{l}\mathbf{u}\mathbf{e};\mathbf{d}\mathbf{e}\mathbf{f}\mathbf{a}\mathbf{u}\mathbf{l}\mathbf{t},1.6\right)$$

where ${S}_{\mathrm{upper}, 0}$ and ${S}_{\mathrm{lower}, 0}$ are *S* values when the values of the pixels in the outer region are set to 0.

### Detection of misassembly using Hi-C

We detected misassemblies in each scaffold using the Hi-C matrix (Additional file 1: Fig. S9). This step was inspired by other Hi-C scaffolding tools, such as 3D-DNA and HiCAssembler [39]. The concept of this function is similar to that of the detection of erroneous edges using Hi-C described in the previous section; however, the implementation is different for handling continuous input scaffolds.

1. We created Hi-C matrices of scaffolds larger than 300 kb and calculated the average number of Hi-C links between bins in the same scaffold for each distance from the diagonal.
2. We created the Hi-C matrix of the target scaffold.
3. We calculated the misassembly score by placing a triangular motif along the diagonal as follows:
  5 $$\mathbf{S}\mathbf{c}\mathbf{o}\mathbf{r}\mathbf{e}=\sum\nolimits_{{\varvec{k}}=1}^{10}\frac{{\left({{\varvec{\mu}}}_{\mathbf{a}\mathbf{l}\mathbf{l},{\varvec{k}}}-{{\varvec{\mu}}}_{\mathbf{t}\mathbf{a}\mathbf{r}\mathbf{g}\mathbf{e}\mathbf{t},{\varvec{k}}}\right)}^{2}}{{{\varvec{\mu}}}_{\mathbf{t}\mathbf{a}\mathbf{r}\mathbf{g}\mathbf{e}\mathbf{t},{\varvec{k}}}}$$

where *k* is the distance from the diagonal, ${\mu }_{\mathrm{target},k}$ is the average value of the pixels at a distance *k* from the diagonal of the target scaffold, and ${\mu }_{\mathrm{all},k}$ is the average value of the pixels at a distance *k* from the diagonal of all scaffolds. If the value of the misassembly score is anomalously high, it suggests that the corresponding bin spans the misassembly (Additional file 1: Fig. S9a).

(4) Peaks were detected in the misassembly score.
(5) We defined *P* as the threshold for the misassembly score peak. If the misassembly score peak is higher than *P*, we surmised that the corresponding bin spans a misassembly. Other Hi-C scaffolding tools used a fixed value for this type of threshold; however, GreenHill automatically determined this threshold. First, we divided the target scaffold at the position of the peak above *P* and calculated *S* at each cut position. Then, we varied *P* from 0 to the maximum of the peaks and determined *P* such that the average of *S* was the maximum (Additional file 1: Fig. S9b).
(6) The bins that have a peak above *P* were surmised as misassembly candidate regions if conditions (3) and (4) were satisfied.

### Detection of erroneous edges using long reads

The edge *e* between nodes *u* and *w* were checked using long read (and PE) information (Additional file 1: Fig. S10).

1. If the number of long read (and PE) links between nodes *u* and *w* exceeded the threshold *minLink* (default: 0), edge *e* was surmised to be the correct edge.
2. We calculated the long read (and PE) links between nodes *u* and *w*’, *u*’ and *w*, and *u*’ and *w*’. If a node pair had links greater than *minLink*, then the edge between the nodes was used instead of edge *e*.
3. Otherwise, misassemblies were detected in the area within 300 kb from the ends of the scaffold based on the mapping information of the long read (and PE). First, we identified the misassembly candidate region using mapping information. For PE and MP, we calculated the start (where the read was mapped) and end positions (the start position plus insert length tolerance) for each read, and calculated the insertion length tolerance using the formula:

6 $$\mathbf{\text{Insert length tolerance}}=\boldsymbol{a}+3\times\boldsymbol{d}$$

where *a* is the average insert size of the library and *d* is the standard deviation of the insert size of the library. For long reads, the starting position was calculated as the end of the alignment region to the scaffold*.* The end position was calculated as the start position plus the distance between the alignment region to the scaffold and the alignment region to the other scaffold. Misassemblies were considered to be located between the start and end positions. Therefore, the region between the start and end positions was detected as the misassembly candidate region. Next, the breakpoint in the misassembly candidate region was determined. Since a drop in coverage indicated a likely misassembly, we divided the scaffold at a minimum coverage point in each misassembly candidate region.

In the presence of misassemblies, we corrected and surmised edge *e* as the correct edge; otherwise, edge *e* was surmised to be an erroneous edge.

### Phasing

In the phasing step, we divided the consensus scaffolds into haplotype blocks and conducted phasing using long read (and PE) and Hi-C read links (Additional file 1: Fig. S11) as follows:

1. We identified the array of haplotype blocks (contigs or scaffolds), which was stored in the merge haplotype step. A phase was represented by a binary value (0 or 1), and each haplotype block consisted of a pair of sequences with phases (haplotype0 and haplotype1). Blocks such as haplotype0 ≠ haplotype1 were identified as haplotype blocks, and blocks such as haplotype0 = haplotype1 were identified as homozygous blocks.
2. Let haplotype_*i*_0 and haplotype_*i*_1 be the sequences of phases 0 and 1 in the *i*-th haplotype block, respectively, where *i* represents an integer. Below, *j* also represents an integer. We calculated long read (and PE) and Hi-C links between haplotype blocks and the number of links supporting the parallel (haplotype_*i*_0 and haplotype_*j*_0 in the same phase) and cross paths (haplotype_*i*_0 and haplotype_*j*_1 in the same phase) between *i*-th and *j*-th haplotype blocks.
3. We connected the pair of haplotype blocks with the largest difference in the number of supporting links between the parallel and cross paths. If the number of links supporting the cross path was larger than that of the parallel path, we swapped the haplotype0 and haplotype1 of one of the haplotype blocks before connecting the pair.
4. We repeated procedure (3) until no pair of haplotype blocks were connected.
5. We divided the scaffold between haplotype blocks that failed to connect. If there was an unphased haplotype block inside a set of connected haplotype blocks, the phase of the unconnected block was determined randomly and the scaffold was not divided.

### Estimation of heterozygosity

Heterozygosity was estimated by *k*-mers (k-length sequences) in whole-genome sequencing reads (Illumina PE for *C. elegans* and cow, HiFi reads for *D. melanogaster*, zebra finch, black rhinoceros, and sterlet, 10X for budgerigar). The *k*-mer in the read was counted, and the *k*-mer histogram was calculated by jellyfish [40]. Genomescope [41] was used to estimate the level of heterozygosity. Results are shown in Additional file 1: Table S1. Notably, the cow sample’s heterozygosity was estimated from previous studies because the model fitting of Genomescope had a large discrepancy with the data.

### Contig assembly

Platanus-allee (v2.2.2-modified; deposited in the repository of GreenHill v1.1.0) was executed using default parameters, except for the input files and multithreading. Particularly, the three commands of Platanus-allee, “assemble,” “divide,” and “phase,” were executed, which corresponded to the contig-assembly, dividing contig at misassembly and phasing modules. The inputs of the “assemble” and “divide” were the Illumina paired-ends, excluding PacBio long reads or Hi-C reads. All libraries were inputted to the “phase” command. For zebra finch, 10X reads were used instead of paired ends.

FALCON-Unzip (v1.2.0 in Bioconda) was executed using the following parameters: (length_cutoff = -1; length_cutoff_pr = 5000; pa_daligner_option = -e0.76 -l1200 -k18 -h70 -w8 -s100; ovlp_daligner_option = -k24 -h1024 -e.95 -l1800 -s100; pa_HPCdaligner_option = -v -B128 -M24; ovlp_HPCdaligner_option = -v -B128 -M24; pa_HPCTANmask_option = -k18 -h480 -w8 -e.8 -s100; pa_HPCREPmask_option = -k18 -h480 -w8 -e.8 -s100; pa_DBsplit_option =—× 500 -s400; ovlp_DBsplit_option = -s400; falcon_sense_option = –output-multi –min-idt 0.70 –min-cov 3 –max-n-read 400 –n-core 24; overlap_filtering_setting = –max-diff 100 –max-cov 150 –min-cov 3 –n-core 24). For cow and zebra finch, FALCON-Unzip assembly was downloaded from NCBI (see “Data availability”). The FALCON-Unzip assembly of zebra finches is the result of running purge haplotig [42] on FALCON-Unzip for removing haplotype duplications in the primary contig set.

Canu (v2.1.1) was executed using the following options: “corOutCoverage = 200 batOptions = -dg 3 -db 3 -dr 1 -ca 500 -cp 50.” The option “corOutCoverage = 200” was set to output corrected reads with sufficient coverage depth. Other options were set for the strict criterion of overlap detection among reads in terms of error rates. Overall, these options were set to avoid the collapse of heterozygous regions and separate haplotypes.

HiCanu (v2.1.1) and Hifiasm (v0.16.1) were executed using default parameters, except for input files and multithreading.

### Execution of FALCON-Phase and Hi-C scaffolding tools

Additional file 1: Fig. S12 shows how the FALCON-Phase and Hi-C scaffolding tools were executed according to the methods in the FALCON-Phase paper [14].

If the input contigs were not generated by FALCON-Unzip, the input contigs were separated into two sequences, purged contigs and haplotigs, using Purge_dups (v1.2.5) [26]. The coverage cutoffs were set manually from the coverage histogram. The headers of the purged contigs and haplotigs were renamed in the format required by FALCON-Phase using an in-house script. Briefly, each haplotigs was mapped to the purged contigs using Minimap2 to find the associated purged contigs. The headers of purged contigs and haplotigs were renamed to indicate the placement.

Input contigs were phased using FALCON-Phase (v1.2.0 in Bioconda) with the following parameters: (min_aln_len = 3000, iterations = 1,000,000, output_format = pseudohap). The parameter for enzyme recognition was set to the recognition corresponding to the Hi-C library (“AAGCTT” for *C. elegans*, “AAGCTT” for *D. melanogaster*, “GATC” for cow and sterlet, “GATC, GAATC, GATTC, GAGTC, GACTC” for zebra finch and budgerigar, and “GATC, GAATC, GATTC, GAGTC, GACTC, CTAAG, CTTAG, CTGAG, CTCAG, TTAA” for black rhinoceros). Two haplotypes named “phased.0” and “phased.1” were generated using FALCON-Phase. For cow and zebra finches, FALCON-Unzip + FALCON-Phase results were downloaded from NCBI (see “Data availability”). This process was performed to increase the accuracy of the following Hi-C scaffolding by creating phased contigs.

Next, we scaffolded phased.0 using two Hi-C scaffolding tools, 3D-DNA and SALSA2.

Scaffolding via 3D-DNA was executed using Hi-C read mapping onto the contigs with Juicer (v1.5.6) [36] using the default parameters. The read-mapping tool was Burrows–Wheeler Alignment (BWA) (v0.7.17-r1188) [43]. The restriction fragment file required by Juicer was generated using the “generate_site_positions.py” script. For multiple restriction enzymes, we generated the BED file using the “digest_genome2.py” script [44] and converted the BED file to the restriction fragment file for Juicer using the “hic-pro2juicer.py” script. Scaffolding via 3D-DNA (v180922) was performed using default parameters.

Scaffolding via SALSA2 was executed using the Hi-C read mapping results from the Arima mapping pipeline (v100617) [37] with BWA, SAMtools (v1.3.1) [45], and Picard (v1.141) [46]. The mapping result in BAM format was converted into a BED file required by SALSA2 using the bamToBed of BEDTools (v2.27.1) [47]. The BED file was used as the input for scaffolding using SALSA2 with default parameters.

Finally, we executed FALCON-Phase again using the pairing of phased.1 and Hi-C scaffolding result as input. The headers of the phased.1 and Hi-C scaffolding results were renamed in the format required by FALCON-Phase using an in-house script. Briefly, each phased.1 contig was mapped to the Hi-C scaffolding results using Minimap2 to find the associated Hi-C scaffolding result. The headers of phased.1 and Hi-C scaffolding results were renamed to indicate the placement. The FALCON-Phase was executed using the same parameters as those in the first FALCON-Phase.

### Creating alignment dot plots

We created the alignment dot plots shown in Fig. 3 using the following method:

1. The scaffolds were aligned to the trio-binned assembly (TrioCanu) with nucmer and the alignment results were filtered using delta-filter.
2. We determined the placement of scaffolds on the trio-binned assembly by aligning them to the trio-binned assembly using Minimap2 with the options of “-c -k 19.” The alignment score was calculated based on the value of the “AS” tag in the PAF file (output of Minimap2). The alignment of a scaffold to the same chromosome in the same direction was chained together. The position of the scaffold was then determined as the alignment position with the largest alignment score.
3. A modified version of the mummerplot was used to create alignment dot plots for chromosome 1 with the alignment results from (1) and placement information from (2).
4. Coloring scaffolds were created using the same method as the “ Coloring scaffolds by haplotypes” section and placed on the left of the dot plots.

Of note, the plot generated by this method is not strictly a “dot plot”, because the mummerplot represents the aligned regions as lines, not dots. However, we refer to it as a "dot plot" in this study, because such a plot was often referred to as a “dot plot” in other articles and the mummperplot document.

### Coloring scaffolds by haplotypes

We color-coded scaffolds according to the corresponding haplotype as follows:

1. The scaffolds (≧ 500 kb) were divided into non-overlapping and fixed-length (100 kb) fragments. The short scaffolds (< 500 kb) were not used for downstream steps.
2. We identified the parental haplotype from which the fragment was inherited. Using the hap-mer information from Merqury, we calculated the number of hap-mers inherited from the mother and father in the fragment. The fragment was identified as being inherited from the parental haplotype with a large number of inherited hap-mers. If the number of inherited hap-mers is the same for the parents, the fragment was identified as homozygous. The fragments were color-coded according to the corresponding haplotype (red for mother, blue for father, and gray for homozygote).
3. We determined the placement of scaffolds on the trio-binned assembly (see “ Creating alignment dot plots” (2)).
4. The fragments were displayed in position on the trio-binned assembly using the placement information from (3) in the color determined by (2).

## Supplementary Information

**Additional file 1: Figure S1.** Haplotype structures of the zebra finch assemblies for all chromosomes. **Figure S2.** Hap-mer blob plots for budgerigar data. **Figure S3.** Hap-mer blob plots for black rhinoceros data. **Figure S4.** Hap-mer blob plots for sterlet data. **Figure S5.** Merge haplotype step. **Figure S6.** Consensus scaffolding using long reads. **Figure S7.** Consensus scaffolding using Hi-C. **Figure S8.** Detection of erroneous edges using Hi-C. **Figure S9.** Detection of misassembly using Hi-C. **Figure S10.** Detection of erroneous edges using long reads. **Figure S11.** Phasing steps. **Figure S12.** Execution of FALCON-Phase and Hi-C scaffolding tools. **Table S1.** Sample information. **Table S2.** Contig assembly statistics. **Table S3.** Knock-out test for evaluation of the characteristic functions of GreenHill. **Table S4.** Resource requirements.

**Additional file 2. **Review_history.

## Data Availability

C. *elegans*: The reference sequences are available in the NCBI genome database (N2: GCF_000002985.6 [48] and CB4856: GCA_000975215.1 [49]). PacBio CLR reads are available in the DNA Data Bank of Japan DRA database (N2: DRR142774 and CB4856: DRR142768 [50]). *D. melanogaster*: The reference sequences are available in the NCBI genome database (ISO1: GCF_000001215.4 [51] and A4: GCA_003401745.1 [52]). Parental short reads are available in the NCBI SRA database (ISO1: SRR6702604 [53] and A4: SRR457665, SRR457666, and SRR457707 [54]). PacBio HiFi, CLR reads are available from https://obj.umiacs.umd.edu/marbl_publications/hicanu/index.html [55]. Cow: Illumina paired-end, PacBio CLR, Hi-C, and parental short reads are available in the NCBI SRA database (PE: SRR6691721–SRR6691727, SRR6691748, SRR6691951–SRR6691953, and SRR6691961, CLR: SRR8224240–SRR8224250, SRR8695274, SRR6691737, SRR6691756–SRR6691758, SRR6691760, SRR6691761, SRR6691781, SRR6691805, SRR6691818, SRR6691819, SRR6691839, SRR6691844, SRR6691846, SRR6691858, SRR6691885, SRR6691887–SRR6691898, SRR6691900, SRR6691916, SRR6691919–SRR6691929, SRR6691945, SRR6691972, SRR6691973, SRR6691976–SRR6691983, and SRR8872908–SRR8872920, and Hi-C: SRR6691720, *B. indicus* short reads: SRR6691719, SRR6691880, SRR6691881, SRR6691906, *B. taurus* short reads: SRR6691901–SRR6691903, and SRR6691907 [56]). Trio-binned parental Canu assemblies are available in the NCBI genome database (GCA_003369685.2 [57] and GCA_003369695.2 [58]). FALCON-Unzip contigs are available in the NCBI genome database (GCA_012069665.1 [59] and GCA_012070425.1 [60]). FALCON-Unzip + FALCON-Phase contigs are available in the NCBI genome database (GCA_012070465.1 [61] and GCA_012070445.1 [62]). Zebra finch: PacBio CLR, HiFi, 10X, Hi-C, parental short reads, and trio-binned parental Canu assemblies are available in the GenomeArk database (https://vgp.github.io/genomeark/Taeniopygia_guttata [63]). FALCON-Unzip contigs are available in the NCBI genome database (GCA_012069585.1 [64] and GCA_012069535.1 [65]). FALCON-Unzip + FALCON-Phase contigs are available in the NCBI genome database (GCA_012069615.1 [66] and GCA_012069575.1 [67]). Budgerigar: PacBio CLR, 10X, Hi-C, and parental short reads are available in the GenomeArk database (https://vgp.github.io/genomeark/Melopsittacus_undulatus [68]). Black rhinoceros: HiFi, Hi-C, and parental short reads are available in the GenomeArk database (https://vgp.github.io/genomeark/Diceros_bicornis [69]). Sterlet: HiFi, Hi-C, and parental short reads are available in the GenomeArk database (https://vgp.github.io/genomeark/Acipenser_ruthenus [70]). The source code of GreenHill is GPL-3.0 licensed, and publicly available from both the Github repository (https://github.com/ShunOuchi/GreenHill [71]) and Zenodo open data repository (https://doi.org/10.5281/zenodo.8041374 [72]).
