# Supplementary material for: GreenHill: a de novo chromosome-level scaffolding and phasing tool using Hi-C
Source: Genome Biol. 2023 Jul 11;24:162. doi: 10.1186/s13059-023-03006-8 (PMC10334647; doi:10.1186/s13059-023-03006-8)
Supplement: Supplementary file 1 — Additional file 1: Figure S1. Haplotype structures of the zebra finch assemblies for all chromosomes. Figure S2. Hap-mer blob plots for budgerigar data. Figure S3. Hap-mer blob plots for black rhinoceros data. Figure S4. Hap-mer blob plots for sterlet data. Figure S5. Merge haplotype step. Figure S6. Consensus scaffolding using long reads. Figure S7. Consensus scaffolding using Hi-C. Figure S8. Detection of erroneous edges using Hi-C. Figure S9. Detection of misassembly using Hi-C. Figure S10. Detection of erroneous edges using long reads. Figure S11. Phasing steps. Figure S12. Execution of FALCON-Phase and Hi-C scaffolding tools. Table S1. Sample information. Table S2. Contig assembly statistics. Table S3. Knock-out test for evaluation of the characteristic functions of GreenHill. Table S4. Resource requirements. [file 13059_2023_3006_MOESM1_ESM.docx]

**Supplementary Material**

**GreenHill: a *de novo* chromosome-level scaffolding and phasing tool**

**using Hi-C**

Shun Ouchi^1^, Rei Kajitani^1^, and Takehiko Itoh^1*^

^1^ School of Life Science and Technology, Tokyo Institute of Technology, 2-12-1 Ookayama, Meguro-ku, Tokyo 152-8550, Japan

*To whom correspondence should be addressed. Takehiko Itoh, Tel: +81-3-5734-3430, Fax: +81-3-5734-3630, Email: takehiko@bio.titech.ac.jp

Figure S1. Haplotype structures of the zebra finch assemblies for all chromosomes.

Each scaffold was color-coded according to the corresponding haplotype. Red, blue, and gray regions correspond to maternal, paternal, and homozygous ones, respectively. The black dashed lines represent boundaries between scaffolds.

Figure S2. Hap-mer blob plots for budgerigar data.

The numbers of hap-mers (parent-specific *k*-mer) from the mother and father are indicated on the x-axis and y-axis, respectively. Each scaffold is represented as a circle. The sizes of the circles indicate sequence lengths. The higher the phasing accuracy, the closer the circles are aligned to the axis. **a.** FALCON-Unzip + GreenHill result; **b.** FALCON-Unzip + 3D-DNA result; **c.** FALCON-Unzip + SALSA2 result.

Figure S3. Hap-mer blob plots for black rhinoceros data.

The numbers of hap-mers (parent-specific *k*-mer) from the mother and father are indicated on the x-axis and y-axis, respectively. Each scaffold is represented as a circle. The sizes of the circles indicate sequence lengths. The higher the phasing accuracy, the closer the circles are aligned to the axis. **a.** Hifiasm + GreenHill result; **b.** Hifiasm + 3D-DNA result; **c.** Hifiasm + SALSA2 result; **d.** Hifiasm Hi-C mode result.

Figure S4. Hap-mer blob plots for sterlet data.

The numbers of hap-mers (parent-specific *k*-mer) from the mother and father are indicated on the x-axis and y-axis, respectively. Each scaffold is represented as a circle. The sizes of the circles indicate sequence lengths. The higher the phasing accuracy, the closer the circles are aligned to the axis. **a.** Hifiasm + GreenHill result; **b.** Hifiasm + 3D-DNA result; **c.** Hifiasm + SALSA2 result; **d.** Hifiasm Hi-C mode result.

Figure S5. Merge haplotype step.

**a.** How to change the input format to paired-haplotype style. We calculate the self-alignment of the input contigs, then compute the opposite contig of each contig. Redundant contigs are removed and the contigs are minced. **b.** Merge haplotype step. A primary-bubble contig and a secondary-bubble counterpart are merged into a consensus contig using bubble information. For non-bubble contigs, contigs with low coverage are considered heterozygous contigs, and contigs with high coverage are considered homozygous contigs. The merging results are stored in array *T*.

Figure S6. Consensus scaffolding using long reads

A schematic diagram for consensus scaffolding using long reads. We construct a scaffold graph, where nodes represent contigs and edges indicate long read links between contigs. Next, we combine the non-branching nodes in which the indegree and outdegree are equal to one. Then, we remove erroneous edges using Hi-C and construct scaffolds.

Figure S7. Consensus scaffolding using Hi-C.

A schematic diagram for consensus scaffolding using Hi-C. First, scaffolds with misassembly are divided using Hi-C contact map and long read coverage information. Next, we construct an undirected graph where nodes represent the ends of scaffolds and edges indicated Hi-C links between them. Then, we check to see if the edge with maximum weight is an erroneous edge, and if so, remove the edge, and if not, connect the nodes to which the edge bridges. This procedure is iterated until the graph no longer changes. The series of procedures are iterated with increasing *L*. Finally, the series of procedures are redone without checking by long read.

Figure S8. Detection of erroneous edges using Hi-C.

A schematic diagram for checking if an edge *e* connecting scaffolds *u* and *v* is an erroneous edge. First, scaffolds *u* and *v* are partitioned into bins of fixed length (100 kb) and a Hi-C contact map is created by counting the number of linking Hi-C read pairs between bins. Then, separation scores *S* and *R* are calculated and used to determine if the edge *e* is an erroneous edge.

Figure S9. Detection of misassembly using Hi-C.

A schematic diagram for detection of misassembly using Hi-C. **a.** A target scaffold is partitioned into bins of fixed length (100 kb) and a Hi-C contact matrix is calculated. We calculate the misassembly score by placing a triangular motif along the diagonal as the difference from average Hi-C links. **b.** *P* is set so that the average of separation score *S* is maximized. The figure shows examples where *P* is 150 (left) and 90 (right). The target scaffold is divided at the position of the peak above *P*, and the average *S* is calculated. In the left example, the scaffold is divided at one location and *S* is 70, so the average *S* is 70. In the right, example, the scaffold is divided at two locations and *S* is 70 and 10, so the average S is 40. Therefore, *P*=150, which has a higher average S, is selected.

Figure S10. Detection of erroneous edges using long reads.

A schematic diagram for detection of misassembly using long reads. **a.** We calculate the orientation with the largest number of long read (and PE) links. If the largest number of links is larger than the *minlink*, we adopt the orientation. Otherwise, we detect misassemblies at the ends of scaffolds.  **b.** Misassembly candidate region is identified based on the mapping information. Then, the position with the lowest coverage in the misassembly candidate region is determined as a breakpoint.

Figure S11. Phasing steps.

A schematic diagram for phasing steps. First, the haplotype blocks are identified. Next, long read (and PE) and Hi-C links between haplotype blocks are calculated. Then, the pair of haplotype blocks with the largest difference in the number of supporting links between the parallel and cross paths were connected. This process is iterated until no pair of haplotype blocks were connected. Finally, the scaffold is divided between haplotype blocks that failed to connect.

Figure S12. Execution of FALCON-Phase and Hi-C scaffolding tools.

A schematic diagram for execution of FALCON-Phase and Hi-C scaffolding tools. **a.** FALCON-Unzip, Canu, HiCanu, and Hifiasm are used as input assembly. **b.** Input contigs except FALCON-Unzip are separated into primary contigs and haplotigs. **c.** Primary contigs are phased using FALCON-Phase. **d.** Phased.0 contigs are scaffolded by Hi-C scaffolding tools. **e.** FALCON-Phase is executed again using the pairing of phased.1 contigs and phased.0 scaffolded as input.

Table S1. Sample information.

| **Species** | **Library type** | **Average read Length (bp)** | **Total length (bp)** | **Expected sequence coverage(×)** | **Heterozygosity (%)** |
| --- | --- | --- | --- | --- | --- |
| *C.elegans* | PE | 250 | 7,943,225,500 | 80 | 0.31 |
|  | PacBio CLR | 10,482 | 8,028,062,739 | 80 |  |
|  | Hi-C | 150 | 5,999,999,890 | 60 |  |
| *D. melanogaster* | PacBio CLR | 17,140 | 30,001,763,138 | 200 | 0.74 |
|  | PacBio HiFi | 24,429 | 5,995,780,057 | 40 |  |
|  | Hi-C | 150 | 8,399,986,576 | 56 |  |
| Cow | PE | 150 | 234,044,926,410 | 86 | 0.65~0.93 |
|  | PacBio CLR | 3,562 | 275,464,947,718 | 100 |  |
|  | Hi-C | 80 | 32,534,720,960 | 12 |  |
| Zebra finch | 10X | 102 | 107,638,065,920 | 105 | 1.47 |
|  | PacBio CLR | 9,025 | 102,219,439,023 | 100 |  |
|  | PacBio HiFi | 11,421 | 39,367,893,111 | 39 |  |
|  | Hi-C | 150 | 95,764,922,700 | 94 |  |
| Budgerigar | 10X | 150 | 97,167,010,234 | 87 | 1.04 |
|  | PacBio CLR | 18,291 | 75,354,296,130 | 67 |  |
|  | Hi-C | 150 | 104,410,326,300 | 93 |  |
| Black rhinoceros | PacBio HiFi | 14,737 | 96,526,923,246 | 32 | 0.21 |
|  | Hi-C | 150 | 226,606,277,700 | 76 |  |
| Sterlet | PacBio HiFi | 13,546 | 111,606,998,728 | 63 | 0.58 |
|  | Hi-C | 150 | 259,186,416,132 | 147 |  |

Summary of sample information used for benchmarks. Heterozygosity was estimated by *k*-mer based method using Genomescope. For cow, heterozygosity from the previous study was used.

Table S2. Contig assembly statistics.

| **Species** | **Assembler** | **Size (bp)** | **Max Length (bp)** | **N50 (bp)** | **QV** | **Switch error rate** |
| --- | --- | --- | --- | --- | --- | --- |
| *C.elegans* | Platanus-allee | 201,915,293 | 2,357,661 | 480,746 | 37.26 | 1.08 |
|  | FALCON-Unzip | 189,700,890 | 4,713,319 | 441,947 | 31.33 | 2.16 |
|  | Canu | 133,218,907 | 2,976,042 | 608,865 | 30.12 | 4.14 |
| *D. melanogaster* | Canu | 296,228,027 | 12,908,400 | 680,260 | 35.12 | 4.16 |
|  | HiCanu | 327,107,205 | 25,974,267 | 9,646,753 | 67.95 | 0.04 |
|  | Hifiasm (p_utg) | 329,314,953 | 12,900,022 | 1,808,138 | 64.55 | 0.03 |
| Cow | Platanus-allee | 5,716,453,794 | 4,343,604 | 446,852 | 31.82 | 6.01 |
|  | FALCON-Unzip | 5,121,908,670 | 99,126,329 | 4,551,535 | 41.84 | 0.18 |
| Zebra finch | Platanus-allee | 2,169,120,089 | 5,688,294 | 470,572 | 35.94 | 0.50 |
|  | FALCON-Unzip | 1,976,041,882 | 20,242,265 | 931,146 | 35.59 | 0.81 |
|  | Canu | 1,928,989,367 | 5,546,997 | 392,153 | 35.34 | 2.09 |
|  | Hifiasm (p_utg) | 2,184,967,032 | 23,325,922 | 3,035,032 | 50.33 | 0.01 |
| Budgerigar | FALCON-Unzip | 2,056,688,110 | 49,947,430 | 4,453,551 | 40.22 | 0.30 |
| Black rhinoceros | Hifiasm (p_utg) | 6,314,336,883 | 9,538,517 | 582,028 | 66.65 | 0.02 |
| Sterlet | Hifiasm (p_utg) | 3,861,485,552 | 10,699,564 | 1,682,164 | 60.74 | 0.02 |

Summary of contig assembly statistics. Size and N50 were calculated for sequences whose length ≥500bp. Size represents the size of assemblies generated by each assembler. QV and switch error rate were calculated by Merqury.

Table S3. Knock-out test for evaluation of the characteristic functions of GreenHill.

| **Species** | **Input contig** | **Kock out (KO)** | **Max Length (bp)** | **N50 (bp)** | **#mis**  **assembly** | **Switch error rate** | **Phasing accuracy** |
| --- | --- | --- | --- | --- | --- | --- | --- |
| *C. elegans* | Platanus-allee | default | 20,808,020 | 17,065,040 | 105 | 1.16 | 0.867 |
|  |  | simultaneous LR-use KO | 18,821,896 | 13,775,982 | 170 | 1.05 | 0.881 |
|  |  | Hi-C-based correction KO | 53,974,611 | 53,973,479 | 90 | 1.17 | 0.825 |
|  | FALCON-Unzip | default | 20,723,971 | 17,142,644 | 108 | 2.15 | 0.801 |
|  |  | simultaneous LR-use KO | 14,847,431 | 8,543,164 | 338 | 2.11 | 0.877 |
|  |  | Hi-C-based correction KO | 35,694,521 | 17,454,434 | 135 | 2.16 | 0.799 |
|  | Canu | default | 19,835,919 | 16,219,706 | 182 | 3.04 | 0.883 |
|  |  | simultaneous LR-use KO | 3,504,707 | 209,411 | 272 | 3.31 | 0.959 |
|  |  | Hi-C-based correction KO | 65,498,193 | 65,498,172 | 209 | 3.01 | 0.887 |
| *D. melanogaster* | Canu | default | 32,550,496 | 25,267,681 | 515 | 4.42 | 0.803 |
|  |  | simultaneous LR-use KO | 30,328,540 | 23,320,938 | 652 | 4.48 | 0.828 |
|  |  | Hi-C-based correction KO | 52,685,516 | 32,655,313 | 543 | 4.45 | 0.809 |
|  | HiCanu | default | 33,217,621 | 24,975,482 | 930 | 0.03 | 0.930 |
|  |  | simultaneous LR-use KO | 27,085,171 | 22,450,516 | 944 | 0.03 | 0.927 |
|  |  | Hi-C-based correction KO | 111,531,930 | 111,531,912 | 931 | 0.03 | 0.917 |
|  | Hifiasm | default | 27,892,039 | 24,570,326 | 742 | 0.03 | 0.920 |
|  |  | simultaneous LR-use KO | 27,007,628 | 22,427,718 | 738 | 0.03 | 0.923 |
|  |  | Hi-C-based correction KO | 49,556,982 | 23,155,838 | 766 | 0.03 | 0.928 |
| Cow | FALCON-Unzip | default | 156,630,926 | 89,758,138 | 7,570 | 0.18 | 0.949 |
|  |  | simultaneous LR-use KO | 146,308,271 | 65,105,278 | 9,863 | 0.17 | 0.971 |
|  |  | Hi-C-based correction KO | 242,373,730 | 103,567,514 | 7,697 | 0.18 | 0.907 |
| Zebra finch | Platanus-allee | default | 152,477,488 | 61,881,567 | 1,332 | 0.57 | 0.953 |
|  |  | simultaneous LR-use KO | 88,255,425 | 33,070,620 | 2,096 | 0.50 | 0.957 |
|  |  | Hi-C-based correction KO | 289,099,635 | 93,668,539 | 1,285 | 0.57 | 0.782 |
|  | FALCON-Unzip | default | 150,748,938 | 70,617,212 | 1,543 | 0.79 | 0.886 |
|  |  | simultaneous LR-use KO | 131,927,695 | 54,233,580 | 2,959 | 0.79 | 0.912 |
|  |  | Hi-C-based correction KO | 393,590,674 | 222,441,212 | 1,615 | 0.78 | 0.669 |
|  | Canu | default | 148,688,728 | 70,920,789 | 2,741 | 2.28 | 0.849 |
|  |  | simultaneous LR-use KO | 92,763,168 | 14,409,374 | 3,972 | 2.34 | 0.900 |
|  |  | Hi-C-based correction KO | 453,243,742 | 262,346,933 | 2,921 | 2.29 | 0.614 |

Summary of Knock-out test result statistics. N50 was calculated for sequences whose length ≥500bp. #misassembly is the number of misassemblies calculated using the reference alignment-based method. Switch error rate were calculated by Merqury. Phasing accuracy represents the proportion of the majority of hap-mers in a scaffold, and a high value of this indicator suggests large-scale haplotype consistency.

Table S4. Resource requirements.

| **Species** | **Input contig** | **Tool** | **CPU time(h)** | **Real time (h)** | **Max memory (GB)** |
| --- | --- | --- | --- | --- | --- |
| *C. elegans* | Platanus-allee | GreenHill | 13.81 | 0.53 | 23.54 |
|  | FALCON-Unzip | GreenHill | 16.67 | **0.74** | 23.18 |
|  |  | FALCON-Phase + 3D-DNA | **10.01** | 1.63 | **17.56** |
|  |  | FALCON-Phase + SALSA2 | 10.90 | 2.03 | 18.48 |
|  | Canu | GreenHill | 20.90 | **1.03** | 24.21 |
|  |  | FALCON-Phase + 3D-DNA | **9.83** | 1.50 | **17.55** |
|  |  | FALCON-Phase + SALSA2 | 10.90 | 2.08 | 18.55 |
| *D. melanogaster* | Canu | GreenHill | **22.73** | **1.02** | 35.41 |
|  |  | FALCON-Phase + 3D-DNA | 26.87 | 2.94 | **18.24** |
|  |  | FALCON-Phase + SALSA2 | 27.41 | 3.29 | 18.80 |
|  | HiCanu | GreenHill | **9.20** | **0.44** | 44.94 |
|  |  | FALCON-Phase + 3D-DNA | 26.90 | 3.68 | **18.25** |
|  |  | FALCON-Phase + SALSA2 | 27.25 | 3.52 | 19.32 |
|  | Hifiasm | GreenHill | **11.49** | **0.45** | 37.04 |
|  |  | FALCON-Phase + 3D-DNA | 26.84 | 3.26 | **18.17** |
|  |  | FALCON-Phase + SALSA2 | 28.03 | 3.50 | 19.03 |
| Cow | FALCON-Unzip | GreenHill | 337.00 | 15.36 | 192.03 |
|  |  | FALCON-Phase + 3D-DNA* | 309.37 | 18.72 | **96.36** |
|  |  | FALCON-Phase + SALSA2* | **285.16** | **13.73** | 97.10 |
| Zebra finch | Platanus-allee | GreenHill | 323.32 | 18.04 | 108.19 |
|  | FALCON-Unzip | GreenHill | 296.51 | 19.41 | 92.06 |
|  |  | FALCON-Phase + 3D-DNA* | **123.25** | **16.37** | 76.32 |
|  |  | FALCON-Phase + SALSA2* | 127.30 | 20.50 | **39.46** |
|  | Canu | GreenHill | 446.53 | 26.00 | 101.89 |
|  |  | FALCON-Phase + 3D-DNA | **201.05** | **22.49** | 154.58 |
|  |  | FALCON-Phase + SALSA2 | 205.86 | 23.77 | **37.09** |
|  | Hifiasm | GreenHill | **102.24** | **7.45** | 89.60 |
|  |  | FALCON-Phase + 3D-DNA | 229.99 | 25.52 | 156.29 |
|  |  | FALCON-Phase + SALSA2 | 209.47 | 24.44 | **50.37** |
| Budgerigar | FALCON-Unzip | GreenHill | 255.34 | 31.30 | 90.52 |
|  |  | FALCON-Phase + 3D-DNA | 240.95 | 41.59 | 108.85 |
|  |  | FALCON-Phase + SALSA2 | **201.35** | **27.58** | **50.53** |
| Black rhinoceros | Hifiasm | GreenHill | **579.32** | **26.80** | 206.37 |
|  |  | FALCON-Phase + 3D-DNA | 9,535.56 | 277.73 | 98.92 |
|  |  | FALCON-Phase + SALSA2 | 8,543.02 | 238.56 | **65.96** |
| Sterlet | Hifiasm | GreenHill | **569.34** | **29.55** | 128.75 |
|  |  | FALCON-Phase + 3D-DNA | 1,300.94 | 71.28 | 80.00 |
|  |  | FALCON-Phase + SALSA2 | 1,242.32 | 79.40 | **65.96** |

Summary of runtime and memory usage. A bold value indicates the best one for each input assembly. *Note that the FALCON-Unzip + FALCON-Phase + Hi-C scaffolding tools runtimes for cow and zebra finch do not include the first round of FACLON-Phase runtime because the results of the first round of FALCON-Phase were downloaded.
