# Supplementary material for: GreenHill: a de novo chromosome-level scaffolding and phasing tool using Hi-C
Source: Genome Biol. 2023 Jul 11;24:162. doi: 10.1186/s13059-023-03006-8 (PMC10334647; doi:10.1186/s13059-023-03006-8)
Supplement: Supplementary file 2 — Additional file 2. Review_history. [file 13059_2023_3006_MOESM2_ESM.docx]

**1^st^ round**

**Reviewer 1**

Chromosome-scale hapotypes are important to study variation in biodiversity and human health.
The authors describe a new method GreenHill for performed phased scaffolding using Hi-C data.
The method performs the error correction and variance-based thresholds and uses iterative approach.
They benchmarked against tools such as Falcon-phase, 3D-DNA and salsa2 for non-human genomes.
The method is available openly and well documented.

The method seems interesting, however there are some comments below:
1) The authors should add a figure to demonstrate the overall method and an example to improve readability.
2) The recent review article must be cited (https://genomebiology.biomedcentral.com/articles/10.1186/s13059-021-02328-9).
3) The authors should include experiments for HiFi and Hi-C datasets that are becoming state-of-the-art datasets. For example, consider VGP or Darwin Tree of Life genomes and datasets.
4) The experiments on phased scaffolding for genomes with different heterozygosity should be presented to improve the strength of the article.

**Reviewer 2**

General comments:

In "GreenHill: a de novo chromosome-level scaffolding and phasing tool using Hi-C", Ouchi et al. present their software tool, GreenHill, a program that improves de novo genome assemblies using long and/or paired read data as well as Hi-C, correcting misassemblies and scaffolding and phasing contigs. The authors tout GreenHill's "versatility," and indeed, by accepting input contigs from multiple types of assemblers (Platanus-allee's homologously paired contigs, FALCON-Unzip's pseudo-haplotypes, or canu's unpaired contigs) and outputting paired chromosome-scale haplotypes, GreenHill does provide an option for finishing genome assemblies to a more complete and useful form, regardless of their initial state.

I had not heard of this program, but was able to download the code, compile it from source, and run the test examples provided without any problems. The program is housed on github, written in C++, and is free software distributed under the GNU public license. The first author responded promptly and helpfully to an issue raised by a GreenHill user. All of this convinces me that GreenHill is a tool that should be publicized so that researchers can test and compare it with other tools using their own data, especially now that, thanks to long read sequencing and excellent assembly methods, "telomere to telomere" diploid genome references are within reach for most organisms.

Unfortunately, the manuscript is still in very rough form, and as a result, GreenHill's algorithm and performance are not as clear as they could be. I believe the manuscript requires major revisions, and my comments are below.

Specific comments:

1. I found no mention of the program's resource requirements (cpu, memory) for different sizes of assembly and read data sets, nor of how they compare to those of other tools. Especially with big projects like VGP spending large amounts of money on compute, this kind of information is very valuable because it informs project costs.
2. Figure 1 is an important way for readers to get a handle on what GreenHill is doing, but the long bars with their red and blue variant points make the figure both complicated and monotonous at the same time. Is there a way to simplify the look by shortening the bars, or maybe just having them be solid red and solid blue? Perhaps borders around the steps would break things up a bit? The whitespace in step b where paired-haplotypes don't need to be compared could be used to show how the homozygous contigs are ordered with the heterozygous pairs. Also "self-alignment" as one step and "mince/merge" as the others is inconsistent. Pick noun or verb and stick with it.
3. In the Results section, the procedure used for running the two other scaffolders is odd. In lines 135-245, the description reads as if you are running FALCON-Phase twice--once before scaffolding and once after. If this is so, it's not clear what the justification is for that. I'm also assuming that FALCON-Phase is not run at all for the GreenHill results. Is this correct?
4. In results tables 1-4, some sort of highlighting or boxing of "optimal" results for each assembler would help for readability.
5. For the cow hapmer blob plots, the red/blue coloring for haplotypes 1 and 2 is not needed, since it is obviously random (as expected). The important thing to show is how large the blobs are and how close they are to the axes, so I would just use one color for them so as not to distract the reader.
6. Sometimes in the text, the authors use wording that makes it unclear whether they are referring to the original assembly or the phased, scaffolded assembly (e.g., line 207). Maybe "input assembly" and "output assembly", respectively?
7. The method used to create the dot plots (mummerplot actually plots line segments, not dots, so maybe change that wording) in Figure 3 should be described in more detail for readers who, like me, don't look at these plots very often. If I'm guessing correctly, it's this: each program outputs "haplotype 1" scaffolds and "haplotype 2" scaffolds, but for any given scaffold, 1 might be Angus and 2 might be Brahman or vice versa. I'm guessing you assigned each output scaffold to Angus or Brahman by some kind of majority rule before plotting it. If that is the case, does every alignment line in the upper left or lower right quadrant indicate a phase switch within a scaffold?
8. The paper's supplemental tables and figures (which I referred to frequently due to lack of information or clarity in the main text) had no captions and were out of order with respect to the rest of the manuscript. This should be corrected.
9. The main text on "consensus scaffolding using Hi-C", especially in lines 430-439, is poorly written and make it difficult, even with Figure S5, to understand what's being done. What does "iterate until the edge disappears" mean?
10. In the description of phasing in the methods and Figure S8, I found the use of the "T" array and the matrices in the figure unhelpful. I would guess that plain language like "haplotype 1" and "haplotype 2", "cross paths" and "parallel paths", would be more clear, since this part of the algorithm is fairly intuitive and doesn't need the mathematical notation for precise description.

**Author’s response**

**Update of GreenHill**

After the first submission of the manuscript, some bugs in GreenHill (version 1.0.0) were discovered and fixed, and the self-alignment method was adjusted for speed, and updated to version 1.1.0. Note that the algorithm design was not modified, and the procedures described in the reviewed manuscript are truly implemented in the new version of GreenHill. The main changes are as follows:

(1) A bug fix in the consensus scaffolding using the Hi-C step.

　The consensus scaffolding using the Hi-C step possibly crashes due to wrongful access to unallocated memory space.

(2) A bug fix in the mapping step.

　This bug may prevent mapping reads using a *k*-mer that matches contigs belonging to the same consensus contig. (see Methods; Mapping reads to contigs).

(3) Improvement of the self-alignment method.

　The self-alignment method for pseudo-haplotype and haplotype-ignorant style inputs was changed to speed up the process. “-P” was previously used as an option for minimap2, but “-D --secondary=no” is used in the new version. Alignment results with < 80% identity are filtered in the new version. Also, the minimap2 version was updated to version 2.24.

**To the Referee 1**

**Comment 1.1**

The authors should add a figure to demonstrate the overall method and an example to improve readability.

**Response**

Thank you for your valuable comment. We had intended to describe the overall flow of GreenHill (from input contigs to output scaffolds), but we realized that it was not easy to understand. We then modified Figure 1 completely to clarify the input and output data, partition its steps, and adopt a more understandable representation of haplotypes. Note that this response also addresses Comment 2.2 of Referee 2 below. Also, to further aid understanding, several additions and corrections have been made in the Methods section.

**Comment 1.2**

The recent review article must be cited

(https://genomebiology.biomedcentral.com/articles/10.1186/s13059-021-02328-9).

**Response**

We appreciate the suggestion and apologize for this oversight. We have now included this article in the Background section as reference No. 4.

**Comment 1.3**

The authors should include experiments for HiFi and Hi-C datasets that are becoming state-of-the-art datasets. For example, consider VGP or Darwin Tree of Life genomes and datasets.

**Response**

Thank you for your valuable comment. As suggested, we downloaded HiFi and Hi-C datasets (zebra finch, black rhinoceros, and sterlet) from the Vertebrate Genomes Project (VGP) and performed additional benchmarks. We have included the additional benchmark results in the revised manuscript (p10 “Zebra finch dataset” and p12 “Other species datasets” in the Result section). These results revealed that GreenHill performs well with the HiFi + Hi-C dataset. We appreciate your feedback, which has helped us to improve the robustness of our proposed approach.

**Comment 1.4**

The experiments on phased scaffolding for genomes with different heterozygosity should be presented to improve the strength of the article.

**Response**

As suggested, we have included data on three additional species (black rhinoceros, sterlet, and budgerigar), and also added a table containing the heterozygosity for each species. (Table S1). The total number of species amounted to seven, and their heterozygosities ranged from 0.21 to 1.4. Additionally, we have included a detailed description of the method used for estimating heterozygosity (p24, “Estimation of heterozygosity” in the Methods section) and provided a discussion of the effect of heterozygosity on the accuracy of genome assembly (p14, in the Discussion section). These additions have significantly improved the strength of the article, and we are grateful for the reviewer’s comment.

**To the Referee 2**

**Comment 2.1**

I found no mention of the program's resource requirements (cpu, memory) for different sizes of assembly and read data sets, nor of how they compare to those of other tools. Especially with big projects like VGP spending large amounts of money on compute, this kind of information is very valuable because it informs project costs.

**Response**

Thank you for your valuable comment. As suggested, we have added GreenHill’s resource requirements and compared them with other tools (Table S4). The corresponding paragraph was also added (p13, “Runtime and memory usage evaluation” in the Result section). GreenHill achieved fewer runtimes for most of the species, and its peak memory usage was less than 206 GB even for the species with a large genome size (> 2Gbp). Consequently, we expect that GreenHill will be able to facilitate projects targeting the genomes of many species.

**Comment 2.2**

Figure 1 is an important way for readers to get a handle on what GreenHill is doing, but the long bars with their red and blue variant points make the figure both complicated and monotonous at the same time. Is there a way to simplify the look by shortening the bars, or maybe just having them be solid red and solid blue? Perhaps borders around the steps would break things up a bit? The whitespace in step b where paired-haplotypes don't need to be compared could be used to show how the homozygous contigs are ordered with the heterozygous pairs. Also "self-alignment" as one step and "mince/merge" as the others is inconsistent. Pick noun or verb and stick with it.

**Response**

Thank you for your valuable feedback on Figure 1. We have made the necessary changes to improve its readability. Major changes are as follows:

(1) we have lengthened the bars with variant points and added boundaries between the steps.

(2) We added a description on the whitespace in step b to show that paired-haplotypes are already paired and classified, and mincing is skipped.

(3) We have changed “mince/merge” to “mincing/merging” for consistency. We are convinced that these changes will make the figure easier for readers to understand.

**Comment 2.3**

In the Results section, the procedure used for running the two other scaffolders is odd. In lines 135-245, the description reads as if you are running FALCON-Phase twice--once before scaffolding and once after. If this is so, it's not clear what the justification is for that. I'm also assuming that FALCON-Phase is not run at all for the GreenHill results. Is this correct?

**Response**

Thanks for your comment. It may seem a bit odd, but we faithfully followed the procedure proposed in the FALCON-Phase paper (Kronenberg *et al.* 2021) by running FALCON-Phase twice, once before Hi-C scaffolding and once after. This was done to obtain phased contigs that can then be scaffolded more accurately with Hi-C data. We have updated the Results and Methods section to clarify that we followed the procedure described in the FALCON-Phase paper and the reason for running FALCON-Phase twice (p6, “Comparison of GreenHill with other approaches”, p 26, “Execution of FALCON-Phase and Hi-C scaffolding tools”). Additionally, we have added a figure to further illustrate the procedure (Fig. S12). The figure shows that FALCON-Phase is first run on the input contigs to generate phased contigs. Hi-C scaffolding is then performed on one haplotype-phased contig. Finally, FALCO-Phase is run again on the scaffolded contigs to phase the final scaffolds. However, you are right about FALCON-Phase not being run for the GreenHill results, because GreenHill has a Hi-C phasing function.

**Comment 2.4**

In results tables 1-4, some sort of highlighting or boxing of "optimal" results for each assembler would help for readability.

**Response**
Thank you for your comment. As requested, we have updated the results in Tables 1–4 to include bold highlighting for the optimal results for each assembler. This change should make the tables easier to read and interpret.

**Comment 2.5**

For the cow hapmer blob plots, the red/blue coloring for haplotypes 1 and 2 is not needed, since it is obviously random (as expected). The important thing to show is how large the blobs are and how close they are to the axes, so I would just use one color for them so as not to distract the reader.

**Response**

We agree that the red/blue coloring is not necessary and that it can be omitted. Instead, we have updated the hapmer blob plots to use a single color to convey the important information (how large blobs are and how close they are to the axes). (Figure 2, Figure 4)

**Comment 2.6**

Sometimes in the text, the authors use wording that makes it unclear whether they are referring to the original assembly or the phased, scaffolded assembly (e.g., line 207). Maybe "input assembly" and "output assembly", respectively?

**Response**

We have revised the manuscript to clarify whether we are referring to the “input assembly” or the “output assembly” (e.g., line 186, line 225, line 259).

**Comment 2.7**

The method used to create the dot plots (mummerplot actually plots line segments, not dots, so maybe change that wording) in Figure 3 should be described in more detail for readers who, like me, don't look at these plots very often. If I'm guessing correctly, it's this: each program outputs "haplotype 1" scaffolds and "haplotype 2" scaffolds, but for any given scaffold, 1 might be Angus and 2 might be Brahman or vice versa. I'm guessing you assigned each output scaffold to Angus or Brahman by some kind of majority rule before plotting it. If that is the case, does every alignment line in the upper left or lower right quadrant indicate a phase switch within a scaffold?

**Response**

Thank you for your comment. According to your request, we have added a new section with a more detailed description of how to create the dot plots (p27-28, “Creating alignment dot plots”). Briefly, each program outputs haplotype1 and haplotype2 scaffolds, but since we do not know which parent each scaffold comes from, we determined the order and orientation of the scaffolds on the maternal and paternal chromosome 1 by mapping scaffolds to the trio-binned assembly, which is assumed to be parental haplotypes, before plotting dot plots.

Also, the upper left or lower right quadrant represents scaffolds, which are color-coded according to their haplotypes. Red, blue, and gray correspond to maternal, paternal, and homozygous regions, respectively. Therefore, the red and blue colors region is switched in the middle in the scaffolds generated by the FALCON-Phase base approach, indicating that a phase switch occurs within a scaffold. To improve readability, we have added an explanation in the caption of Figure 3.

We agree that the plot generated by mummperplot is not strictly a "dot plot." However, its plot was often referred to as a "dot plot" in other articles and mummerplot documents, and we did not find another suitable name. We still used "dot plot" in the revised manuscript, but the note about the notation was added at the end of the section above.

**Comment 2.8**

The paper's supplemental tables and figures (which I referred to frequently due to lack of information or clarity in the main text) had no captions and were out of order with respect to the rest of the manuscript. This should be corrected.

**Response**

We apologize for the mistakes and thank the referee for pointing them out. As suggested, we have added captions and corrected the order of the supplemental tables and figures.

**Comment 2.9**

The main text on "consensus scaffolding using Hi-C", especially in lines 430-439, is poorly written and make it difficult, even with Figure S5, to understand what's being done. What does "iterate until the edge disappears" mean?

**Response**

Thank you for your comment. As requested, we have modified the description of “consensus scaffolding using Hi-C” to make it easier to understand what’s being done. The phrase “iterate until the edge disappears” means to iterate until the graph no longer changes. The notation and colors in Figure S7 (previous S5) were also modified for clarity.

**Comment 2.10**

In the description of phasing in the methods and Figure S8, I found the use of the "T" array and the matrices in the figure unhelpful. I would guess that plain language like "haplotype 1" and "haplotype 2", "cross paths" and "parallel paths", would be more clear, since this part of the algorithm is fairly intuitive and doesn't need the mathematical notation for precise description.

**Response**
 Thank you for your comment. We realized that the representations of phases (binary values) and haplotype blocks were not clearly distinguished in the previous manuscript. As suggested, we revised the "Phasing" section under Methods. The notation of *T* has been replaced with haplotype0 and haplotype1, and several sentences have been revised. Additionally, corresponding Figure S11 (previous S8) has been modified like Figure 1 (overview of GreenHill) for clarity.

**2^nd^ round**

**Reviewer 1**

The authors have addressed our comments, thank you!

**Reviewer 2**

I have reviewed the revised manuscript and am satisfied that my concerns have been addressed.
